# Association of Betel Nut with Carcinogenesis: Revisit with a Clinical Perspective

**DOI:** 10.1371/journal.pone.0042759

**Published:** 2012-08-13

**Authors:** Rajeshwar N. Sharan, Ravi Mehrotra, Yashmin Choudhury, Kamlesh Asotra

**Affiliations:** 1 Radiation and Molecular Biology Unit, Department of Biochemistry, North-Eastern Hill University, Shillong, Meghalaya, India; 2 Division of Cytopathology, Center for Oral Cancer and Precancer, Moti Lal Nehru Medical College, Allahabad, Uttar Pradesh, India; 3 Department of Biotechnology, Assam University, Silchar, Assam, India; 4 Ganeshiva Consultants, Los Angeles, California, United States of America; Ohio State University, United States of America

## Abstract

Betel nut (BN), betel quid (BQ) and products derived from them are widely used as a socially endorsed masticatory product. The addictive nature of BN/BQ has resulted in its widespread usage making it the fourth most abused substance by humans. Progressively, several additives, including chewing tobacco, got added to simple BN preparations. This addictive practice has been shown to have strong etiological correlation with human susceptibility to cancer, particularly oral and oropharyngeal cancers.

The PUBMED database was searched to retrieve all relevant published studies in English on BN and BQ, and its association with oral and oropharyngeal cancers. Only complete studies directly dealing with BN/BQ induced carcinogenesis using statistically valid and acceptable sample size were analyzed. Additional relevant information available from other sources was also considered.

This systematic review attempts to put in perspective the consequences of this widespread habit of BN/BQ mastication, practiced by approximately 10% of the world population, on oral cancer with a clinical perspective. BN/BQ mastication seems to be significantly associated with susceptibility to oral and oropharyngeal cancers. Addition of tobacco to BN has been found to only marginally increase the cancer risk. Despite the widespread usage of BN/BQ and its strong association with human susceptibility to cancer, no serious strategy seems to exist to control this habit. The review, therefore, also looks at various preventive efforts being made by governments and highlights the multifaceted intervention strategies required to mitigate and/or control the habit of BN/BQ mastication.

## Introduction

*“They are always chewing Arecca, a certaine Fruit like a Peare, cut in quarters and rolled up in leaves of a Tree called Bettre (or Vettele), like Bay leaves; which having chewed they spit forth. It makes the mouth red. They say they do it to comfort the heart, nor could live without it.”— Pigafetta, in Purchas,i. 38. [Circa 1521]* [1]

The above quotation gives a vivid description of the *Areca* nut (AN), which is more commonly referred to as betel nut (BN). BN is masticated or chewed either alone or in combination with a wide variety of additives, which are often wrapped in the leaf of *Piper betle* (popularly called as betel leaf), giving it the more common name, betel quid (BQ) [2], [3]. More significantly, the verse also alludes to the rampant use of BN or BQ as a masticatory product and its probable addictive nature.

**Figure 1:**
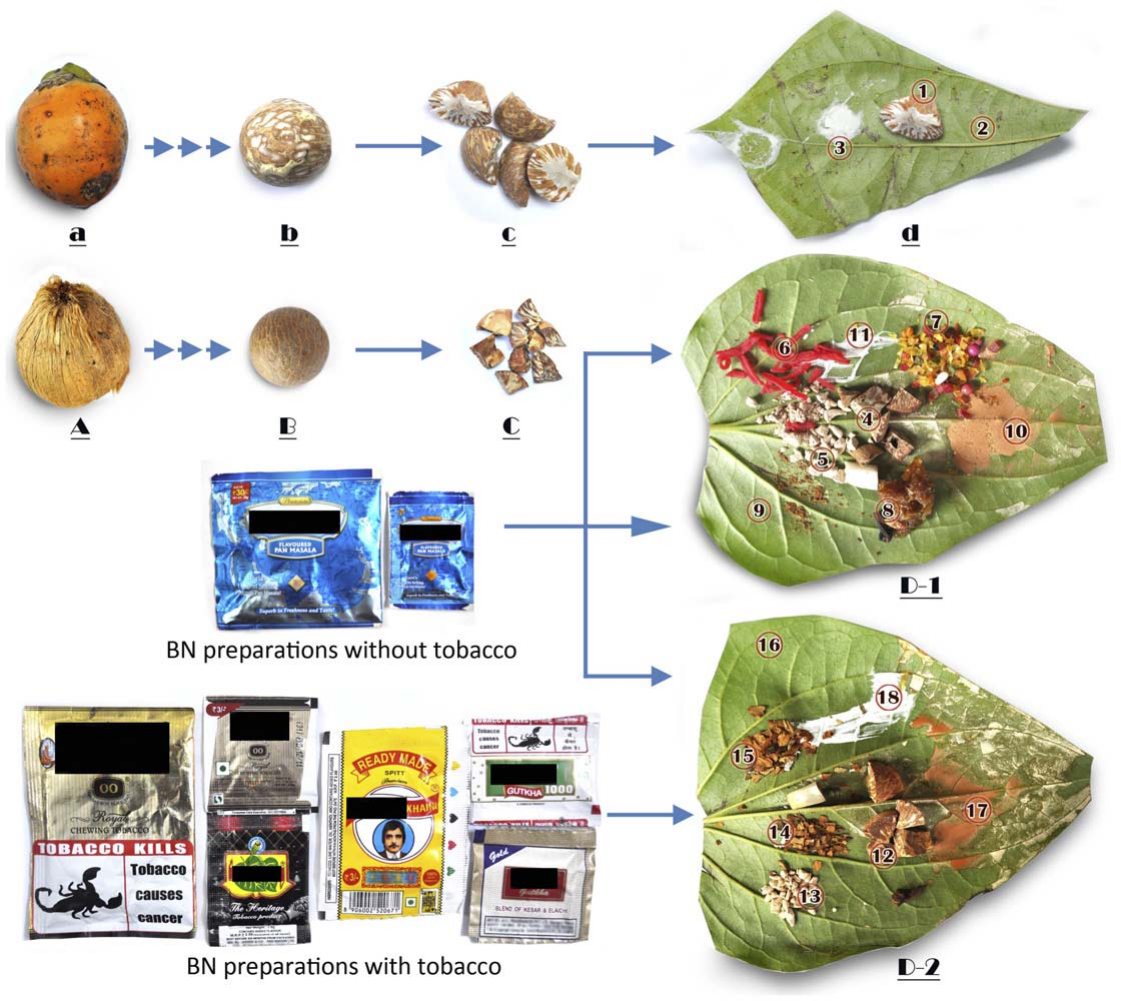
Betel nut (BN), betel quid (BQ) and different preparations associated with its mastication, including their commercial reincarnations. The unripe *Areca* fruit (a), either directly or after short curing is shelled to get wet and soft BN (b) (*tambul* or *kwai*), which after cutting into 4–5 pieces (c & 1) is normally consumed with a piece of betel leaf (2) and slacked lime (3) making a simple BQ (d). The ripe *Areca* fruit (A), after drying and curing is shelled to get dry and hard nut (B), which is cut into smaller pieces (C) (*supari*) for mastication. The dry pieces of BN (4 & 12) are usually masticated with a variety of additives (5–8), all of which usually contain BN, on a betel leaf (9) supplemented with catechu (10) and slacked lime (11) in a complex BQ (D-1). A variant of the complex BQ (D-2) may include all of the above plus a variety of chewing tobacco additives (13–15). Commercialization of this widespread practice of BQ mastication has lead to mushrooming production of convenient and inexpensive alternate forms of BN preparations without (*paan masala*) or with tobacco (*gutkha*). Few of these products, packages in sachets (shown) or containers of various sizes (not shown), which are widely available in markets in India are shown here. All these products have no standardized production frame or declaration of nutritional components. See text for details.

BN is normally harvested as unripe (yellow-green) or ripe (orange/red) fruit from the tropical palm, *Areca catechu*. The *Areca* fruits may be sun dried for several weeks, fibrous shells removed and the hard, dry nuts, commonly called ‘*supari*’ in India, are ready for use. Such sun dried variety of BN is very hard, and is cut into small pieces to make it easier to masticate. Alternatively, the ripe BN are boiled for several hours in an aqueous solution containing the bark of the plant *Eugenia jambolana*, jaggery or brown sugar, and various edible oils, to ‘cure’ it. The cured fruits are sun dried for several weeks, fibrous shell removed and very hard, brown nuts, another variety of ‘*supari*’, are ready for use. In contrast, ripe, partly ripe or unripe *Areca* fruits are freshly picked, fibrous shells removed and the relatively soft nuts are ready for mastication (Figure 1). Occasionally, the fruits can be cured by burying them into moist pits for 1–2 weeks for fermentation (maturation) before shelling and use. Such raw and wet variety of BN, widely used particularly in the northeastern part of India, is locally called ‘*kwai*’ or ‘*tambul*’. These are relatively soft, and hence, larger pieces of the nut are masticated. Aged people may, at times, even masticate powdered form of the raw/wet or dry variety of BN [2]–[5].

The BN is consumed either alone or as BQ in which case it is wrapped in a betel leaf along with slaked lime (Calcium oxide and Calcium hydroxide) and additives (Figure 1). The BQ with a variety of additives is commonly referred to as ‘*paan*’ in India. The components of BQ can vary widely between countries, regions, communities and individuals. However, the major constituents are BN, catechu (*Acacia catechu*), a resinous extract from the wood of the *Acacia* tree, slaked lime, various additives, such as grated coconut, aniseed, pepper mint, cardamom, cloves, perfumes and stimulants wrapped in betel leaf (Figure 1) [2]–[5]. In India, most habitual chewers of BQ add tobacco, while in some countries, such as Papua New Guinea and China, tobacco is normally not added [6]. In northeastern India, *kwai* or *tambul* is primarily consumed only with betel leaf and slaked lime (Figure 1) [5]. Betel leaf is perishable and the preparation of BQ is somewhat complex. Hence, over the past few decades, commercial BQ substitutes, a flavored and sweetened dry mixture of BN, catechu and slaked lime either with tobacco (*gutkha* or *khaini*) or without tobacco (*paan masala*), have become increasingly popular [6]. These products are packaged in small, attractive and inexpensive sachets, and are easily advertised and marketed, often claimed to be safe products (Figure 1). Use of *gutkha* often begins at a very young age. *Gutkha* contains large amounts of sweeteners to conceal the bitterness of tobacco, and children often consider it as a type of candy. Many people take *gutkha* to be harmless and mere ‘mouth freshener’ [7]. *Gutkha* and *paan masala* are consumed by very young and old alike, particularly in India, and also among migrant populations from these areas worldwide [6]. It has been reported that in the Hunan province of China, the shell of the *Areca* fruit is not removed before consumption. Three main variants of BQ are prepared - husk without BN (kernel, seed, endosperm) being the most common, husk with other substances e.g. dried grapes, and husk with BN [8].

Several studies have reported the effect of BN and its constituents on human health, especially as a possible cause of oral cancer (OC) [2]–[5], [9]. This review focuses on the consequences of BN chewing with a clinical perspective and seeks to bring into perspective the strategies required as well as those adopted by different countries in order to curb the growing hazards supposed to be ensuing from their usage.

## Methods

### 1. SEARCH STRATEGY

**Figure 2:**
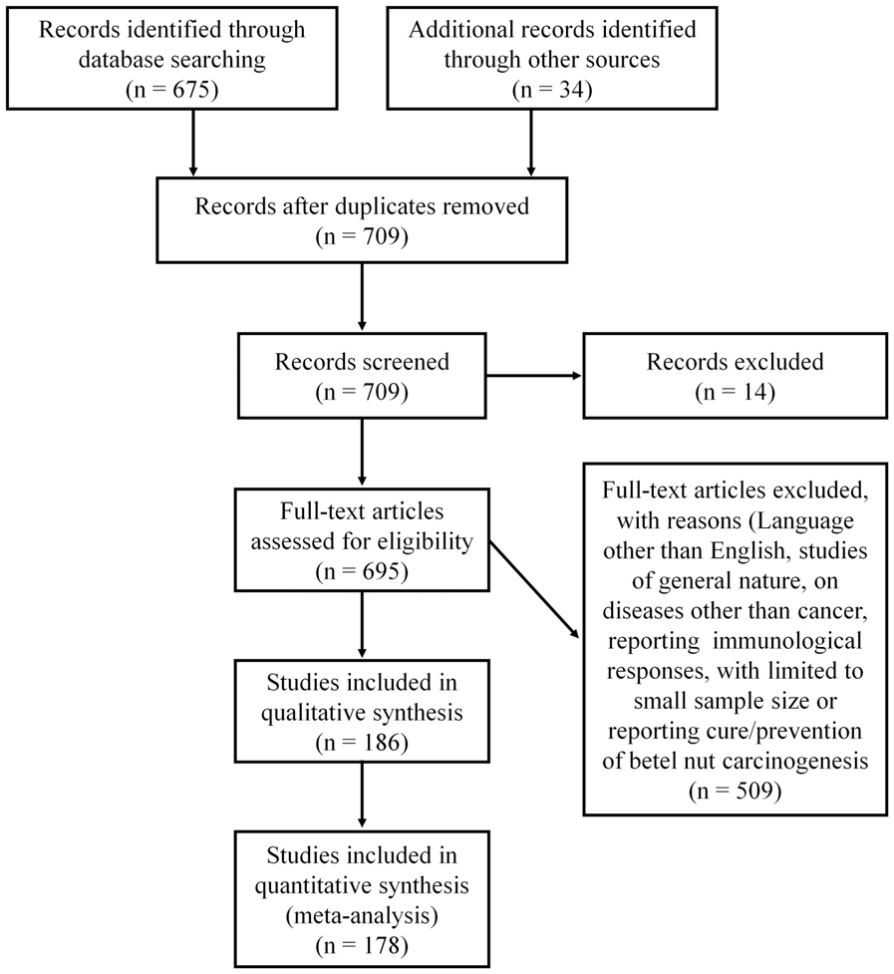
Flow chart of included studies. The flow chart depicts the number of citations and resource materials that have been screened, excluded and/or included in the systematic review.

The studies included in this review have been retrieved from the PUBMED database of the National Library of Medicine, National Institutes of Health, Bethesda, Maryland, by setting limits for papers published only from 1985 to 2012, using the keywords “Betel nut AND cancer”. The search was refined using the operator ‘AND’ in order to retrieve the desired results. This search yielded 675 records (see Figure 2; Flow Chart of included studies).

### 2. EXCLUSION AND INCLUSION CRITERIA

Criteria for exclusion were reports in languages other than English, reports for which abstract was not available, studies pertaining to association of *Areca* with studies other than cancer, studies which investigated the association between cancer or cancer risk with other factors such as tobacco chewing, smoking and alcohol consumption only, without considering BN exposure, studies which focused on single or limited cases, studies which delved into the immunological responses to BN chewing, studies which investigated the treatment of OC caused by BN chewing, and studies of general interest featuring BN without showing a clear role of BN in the pathogenesis of cancer/pre-cancerous conditions. Ultimately, a total number of 148 reports indexed in PUBMED were found to satisfy the criteria for inclusion. Several pertinent reports not indexed in PUBMED were obtained by manual searches, and 30 such reports which satisfied the criteria for inclusion were further retrieved. Thus, the total number of reports included in this review is 178 (see details in Figure 2; Flow chart of included studies).

## Results

### 1. HISTORY OF BETEL NUT AND BETEL QUID USAGE

The earliest use of BN as a masticatory object by humans has been mentioned by Theophrastus in scripts dating around 430 BCE (Before Common Era), which described it as a component of the betel morsel. Chinese texts of 150 BCE, also mention BN as “*pinlang*”. In Persia (modern Iran), it is believed that around 30,000 shops sold BN in the capital town during the reign of Khosrau II, the King of Persia during 590–628 AD. The use of BN by humans has been known since the 4^th^ century AD in different parts of the world, including the South and South-East Asia, several Pacific islands, many regions of the former Soviet Union, parts of North America and Europe [2], [5], and is deeply ingrained in many socio-cultural and religious activities [2]–[5]. A study of the ‘Skull from Bangkok’ collected by Rudolf Virchow (Berlin, Germany) in the late 19th century, as part of an extensive anthropological collection of skeletons and skulls from all over the world, shows brown black stains because of BQ chewing, on the few remaining teeth of the maxilla. In fact, an extensive number of skulls from the South- and Southeast Asia in the collection have been found to show similar betel stains. The Skull from Bangkok is a proof that BQ chewing was prevalent in Siam of the late 19th century [10]. BN is used by both men and women though in some societies the latter predominate, across all age groups, and social classes [3]. In fact, only three other ‘addictive’ substances, namely nicotine, alcohol and caffeine, are used more widely than BN/BQ in the world today [11].

### 2. DEMOGRAPHICS

It is estimated that currently 10% of the world population or nearly 700 million individuals might be consuming BN in different forms across the globe [2], [4], [5]. Epidemiological surveys show that in the past 2 to 3 decades, 20–40% of the population in India, Nepal and Pakistan have used BQ. India has the largest BN chewing population in the world. In fact, aggressive marketing, easy availability and reasonable price of *gutkha* have made it very attractive to youth, and an alarmingly high number of children and teenagers in India. According to one estimate, as many as one in three individuals regularly or occasionally chew *gutkha* [7]. Although a decreasing trend in the consumption of BQ has been observed in certain countries or regions, such as in Thailand, an alarmingly high chewing prevalence has been found among the Palauans of the West Pacific (72–80% use of BQ, with 80% found to be consumers of tobacco-added mixtures) [12]. In China, BQ chewing is largely prevalent in the Hunan province [8]. An inter-country Asian Betel-quid Consortium study (the ABC study) was conducted for East Asia: Taiwan, Mainland China, Malaysia, Indonesia and South Asia: Nepal and Sri Lanka [12]. Chewing rates among men (10.7–43.6%) were significantly higher than women (1.8–34.9%) in Taiwan, Mainland China, Nepal and Sri Lanka, while women's rates (29.5–46.8%) were higher than that in men (9.8–12.0%) in Malaysia and Indonesia. An emerging, large group of new users has been identified in Hunan province in the Mainland China (11.1–24.7%), where chewers have the unique practice of using the dried husk of *Areca* fruit rather than the solid nut used by others. Although the raw *Areca* used in Hunan province is imported from Hainan in Mainland China as well as from Thailand, the vast majority of BQ products in China, including commercial forms, are manufactured locally. The Xiangtan city in Hunan province, where BQ chewing was reported to be very common (prevalence 64.5–82.7%), is also where most BQ production factories and workshops are located. The prevalence of BQ chewing in Hunan men was higher in the younger age groups, suggesting that Hunan province is an emerging region of BQ usage. The improving economy and easy access to BQ products there, supplemented by aggressive advertising campaigns, could be the factors responsible for the widespread use of this substance, particularly among young people. Men in the Eastern and South Asian study communities were deemed likely to combine chewing with smoking and drinking (5.6–13.6%). Low level of school education, alcohol drinking and tobacco smoking were identified as factors associated with BQ chewing [12].

South Asian immigrants in Australia, Europe, the United Kingdom, South and East Africa and the Malay Peninsula continue using BN products, including *paan* and *gutkha*, long after immigration [7]. The United Kingdom is the leading importer of *gutkha* outside of Asia, with imports having doubled in the last three decades. The sale and use of *paan* and *gutkha* are legal in the United States and they are readily available in ethnic enclaves, widely used by the large and rapidly growing South Asian communities [7].

### 3. CONSTITUENTS OF BETEL NUT AND ITS ACTIVE PRINCIPLES

**Figure 3:**
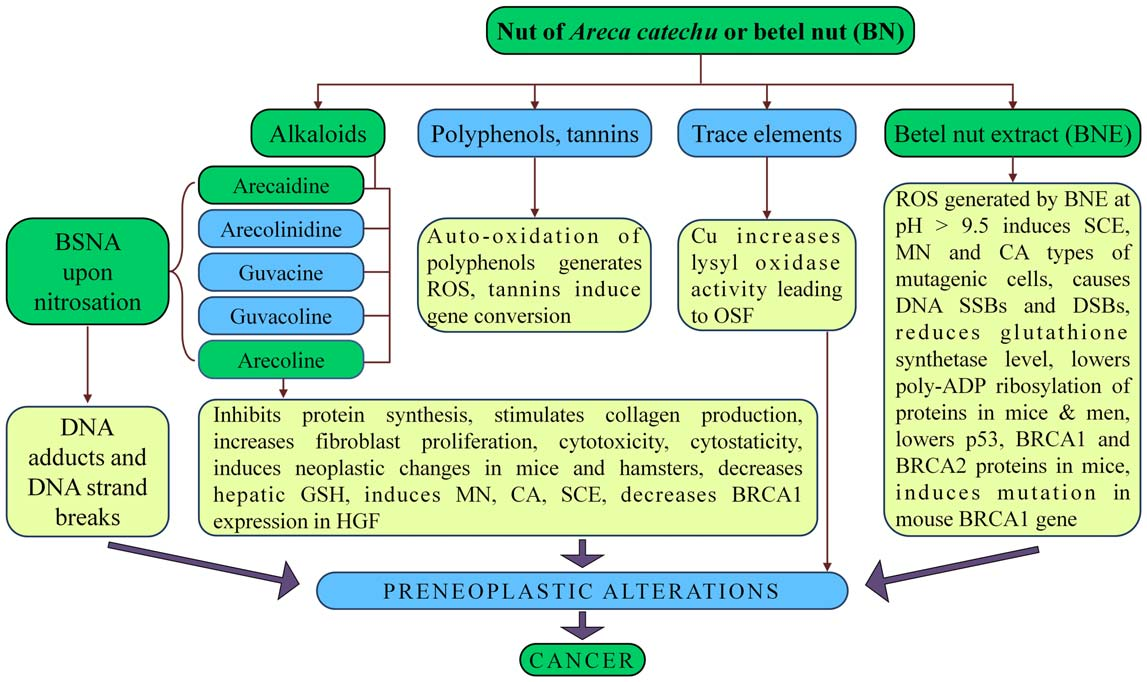
Simplified flow chart of main events of BN induced carcinogenesis. The simplified flow chart is intended to highlight the complexity of BN and its constituents, and how they affect different metabolic components and systems of a cell to eventually lead to carcinogenic transformation. For more details see reviews in references 2–5.

The constituents of BN include crude fiber, carbohydrates, fats, polyphenols, alkaloids, tannins, proteins and water. Trace amounts of fluorine, sapogenins (glycosidic derivatives of steroids and triterpenoids) and free amino acids have also been reported in some forms. The relative amounts of these constituents are highly variable in produce of different regions as well as in the dry or raw/wet variety of BN. Geographical and climatic conditions of growth of the *Areca* palm tree and the methods of curing BN are main factors that contribute to the observed variation in the constituents [5]. The raw and wet variety of BN is relatively rich in all constituents as compared to the dry variety [2], [5]. Notwithstanding these variations, the active components of both forms of BN, which produce BN associated effects, are primarily the alkaloids, polyphenols, and tannins (Figure 3). Figure 3 also highlights the outlines of the main events triggered in a living cell upon exposure to BN and/or its components that eventually lead to carcinogenic transformation of the cell (for details see reviews 2–5).

#### (a) Alkaloids

Alkaloids are reduced pyridines. BN contains several alkaloids, of which arecoline and arecaidine are biologically highly relevant. Arecoline (1,2,4,5-tetrahydro-1-methyl-pyridinecarboxylic acid; molecular weight 155.19 Da) is the most abundant alkaloid of BN followed by arecaidine (1,2,5,6-tetrahydro-1-methyl-3-pyridinecarboxylic acid; molecular weight 141.17 Da). Other alkaloids, such as guvacine (methyl ester of arecaidine), guvacoline (methyl ester of guvacine) and arecolinidine are also present in small to very small or trace amounts [5]. The amount of alkaloid in BN varies with seasonal and geographical variations. In an aqueous extract of Taiwanese BQ composed of fresh BN, betel inflorescence and red lime paste (80.5∶12.5∶7 by weight), arecaidine was the most abundant alkaloid (7.53 mg/g dry weight) and guvacoline the least abundant (0.26 mg/g dry wt.). Cold storage or freeze drying does not bring about alterations in the amount of alkaloids. However, arecoline content is reduced variably following processing of the nut by different methods in different regions of the world. The alkaloids may be converted to several derivatives, each of which can potentially produce even more diazohydroxide derivatives. Presence of most of these derivatives has been demonstrated in the saliva of BQ chewers [4], [9], [13].

#### (b) Polyphenols

Catechin, flavanoids, flavan-3:4-diols, leucocyanidins and hexahydroxyflavans are the prominent polyphenols found in BN [2], [5]. Huang *et al.* have reported that betel nut extract (BNE) contains catechin based procyanidins which range from dimers to decamers and polymers [14]. During mastication, either as BN or BQ, they get oxidized and confer the characteristic red color to saliva, teeth and lips of BN/BQ masticator.

#### (c) Tannins

Specific types of polyphenols that are capable of precipitating proteins are tannins. The predominant tannin of BN is gallotannic acid, which is present in the outer part of the nut. In addition, minor amounts of gallic acid, D-catechol and phiobatannin are also present in the inner part of the nut [2], [5].

#### (d) Trace elements

BN and *paan masala* have been reported to contain sodium, magnesium, chlorine calcium, vanadium, manganese, copper and bromine. The copper content in samples of raw and processed BN was analysed and reported to be much higher than that found most frequently in other nuts consumed by humans. The mean concentration of copper in samples of processed, commercially available BN was 18±8.7 µg/g. In an Indian Food Report, the copper content of processed BN was found to be 2.5 times that of the raw BN [9].

#### (e) Reactive oxygen species

Cellular metabolism of BN or BQ components may also generate reactive oxygen species (ROS), such as superoxide anion radicals (O_2_^.−^) and hydrogen peroxide (H_2_O_2_) at pH greater than 9.5 [15]. While saliva was found to inhibit both O_2_^.−^ and H_2_O_2_ formation from BQ ingredients, ROS are formed in the alkaline chewing mixture within the saliva of a chewer due to the addition of slaked lime [16], [17].

### 4. GENERAL EFFECTS AND ADDICTIVE POTENTIAL OF BETEL NUT

In old Indian scripts such as *Vagbhata* (4^th^ century), and *Bhavamista* (13^th^ century), BN has also been described as a ‘therapeutic agent’ [5]. BN users report increased well-being and stamina, a soothing effect on the digestion, protection of the mouth and gums, and some euphoria. Its use was recommended in wide ranging human diseases and other disorders, which included vitiligo or leucoderma, leprosy, anemia, digestive disorders and infections, urinary and dental infections as well as obesity. BN is also reported to have aphrodisiac property and has been recommended as a general stimulant. In China, it has been used as a vermifuge since the 6^th^ century [5].

BQ chewing has been claimed to produce a sense of well-being, euphoria, warm sensation of the body, sweating, salivation, palpitations, heightened alertness, improved concentration and relaxation, diminished hunger, improved digestion and an increased capacity to work [2], [18]. BN is also reported to have varied and widespread stimulating effects [2], [19], [20]. Small scale studies suggest that BN use may result in a dependence syndrome, though large scale studies testing this hypothesis are lacking [21], [22]. Using a modified version of the Fagerstrom Test for nicotine dependence, it was found that 7% of the patients exhibited one of the following characteristics: daily chewing, chewing within one hour of awakening, difficulty in avoiding chewing, and increasing the quantity chewed. Patients with these symptoms are categorized as ‘severe’ or ‘heavy’ users of BN. Winstock *et al.* reported that 10 out of 11 current and former heavy BN users reported cessation withdrawal effects with the mean severity of Dependence Score of 7.3 consistent with the existence of a dependence syndrome among those who use BN products [22]. However 55% among them used tobacco and BN in combination. Benegal *et al.* reported that about two out of five persons using BN preparations without tobacco additives developed a recognizable pattern of dependent use, satisfying both Diagnostic and Statistical Manual of Mental Disorder, 4^th^ edition (DSM-IV; 38.8% of BN users) as well as International Classification of Diseases, 10^th^ revision (ICD-10; 40.8% of BN users) criteria for current dependence [23]. Given the addiction potential of nicotine, the prevalence of dependence among those using BN preparations with tobacco additives was much higher than among persons using BN alone. Their findings provide support for the concept of an identifiable BN dependence syndrome, which can be diagnosed using criteria very similar to the ones currently used for other substances of abuse.

### 5. BETEL NUT CONSUMPTION AND ORAL MALIGNANCY

**Figure 4:**
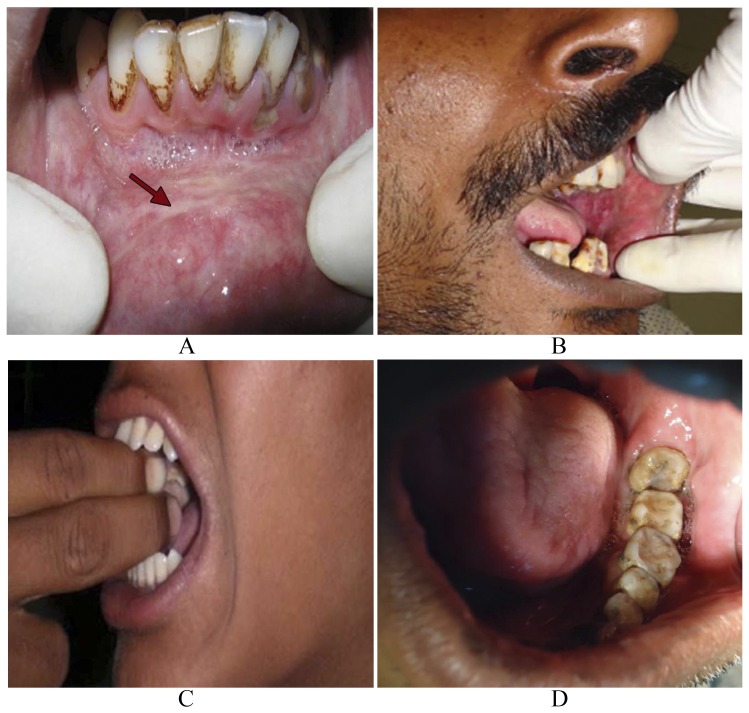
Clinical conditions associated with BN mastication. Mastication of BN/BQ, even without tobacco, manifests itself in some preneoplastic alterations in the oral cavity of the masticator. This includes appearance of whitish patches or plaque (arrow) in the buccal mucosa, known as oral leukoplakia (OL) (A), or its variant with reddish patches/plaques, known as oral erythroplakia (OE) (B). In a third clinical manifestation, stiffening of oral mucosa leads to a clinical condition known as oral submucous fibrosis (OSF) characterized by inflammation and reduced fibro-elasticity which limits the opening of the mouth (C). Prolonged usage lead to a typical clinical manifestation known as the betel chewer's mucosa (BCM). This clinical condition is characterized by brownish-red discoloration of the oral mucosa, especially found in elderly BN chewing women (D).

Prolonged as well as excessive usage of BN has been reported to exert significantly adverse effects on human health. There is enough evidence to suggest that BN products, even without tobacco, are associated with increased risk for the development of oral malignancy, such as oral squamous cell carcinoma (OSCC). A vast majority of BN users show precancerous clinical conditions, such as oral leukoplakia (OL) (Figure 4A) as well as its variant, oral erythroplakia (Figure 4B) or oral submucous fibrosis (OSF) (Figure 4C) among others. The risk is reported to be higher for *paan masala* chewers [24].

**Figure 5:**
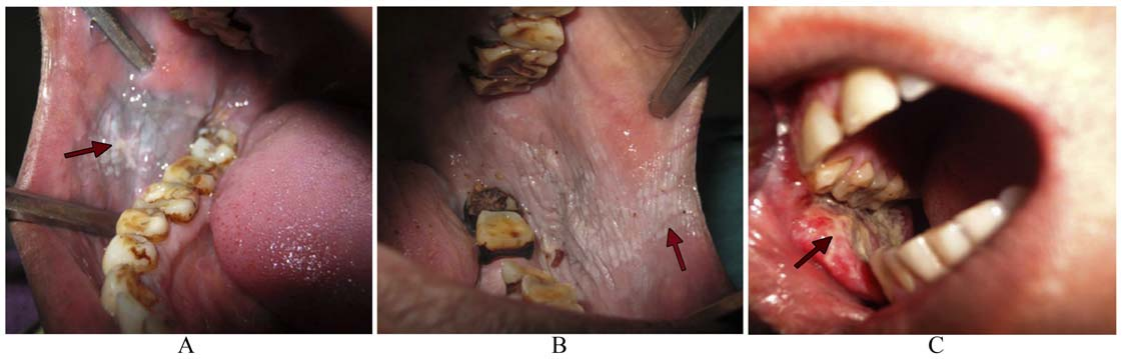
Potentially malignant and malignant conditions associated with BN mastication. Prolonged mastication of BN/BQ eventually manifest itself in development of cancerous condition in the oral cavity of the masticator. Potentially malignant lesions in the oral cavity include lichenoid lesion(s) in the cheek (arrow) close of the site of mastication (A) or even tongue (not shown). At a late stage, lichenoid lesions lead to formation of Oral lichen planus (OLP), which is a type-IV contact hypersensitive type of potentially malignant lesion seen in the oral cavity of BN chewers (arrow) (B). A patient with history of prolonged use of BN alone (without tobacco) eventually shows development of a cancerous condition clinically known as Oral squamous cell carcinoma OSCC (arrow) in his right cheek (C), which was the primary site of BN mastication.

*In vitro* studies have demonstrated that BN extracts containing arecoline inhibit growth and protein synthesis in cultured human periodontal fibroblasts. These findings suggest that BN may be cytotoxic to periodontal fibroblasts and may exacerbate preexisting periodontal disease as well as impair periodontal reattachment [24]. The use of BQ was also found to be associated with the appearance of lichenoid lesions on the buccal mucosa and, occasionally, on the tongue (Figure 5A). These lesions are found at the site of quid placement in BN chewers. Fine wavy keratotic lines are seen to radiate from a central red/atrophic area and the keratotic striae are parallel to each other. The histology is suggestive of a lichenoid reaction and the lesion is noted to resolve following cessation of BN use. Thus, such lichenoid lesions are considered to be type-IV contact hypersensitivity-type lesions, which clinically resemble oral lichen planus (OLP) (Figure 5B) [24].

Another condition associated with prolonged BQ use, especially among elderly women, is betel chewer's mucosa (BCM), which is characterized by a brownish-red discoloration of the oral mucosa (Figure 4D). BCM is often accompanied by encrustation of the affected mucosa with quid particles, which are not easily removed and exhibit a tendency for desquamation and peeling [24]. Several epidemiological studies have shown that the prevalence of BCM varied widely between 0.2% in a Cambodian population in 1995 to 60.8% in the same population in 1996, while a prevalence of 21.9% was reported in a Malaysian population in 1995. Histologically, BCM shows epithelial hyperplasia, which is encrusted with an amorphous deposit. This reacts positively to von Kossa staining suggesting that these granules, which are both intra- and intercellular, may contain calcium from the calcium hydroxide of slaked lime. The presence of the human papilloma virus (HPV) subtypes 11, 16 and 18 have also been demonstrated in BCM but the significance of this is not fully understood. At present BCM is not considered to be potentially malignant, although the condition often coexists with other mucosal lesions such as OL (Figure 4A), OE (Figure 4B) and OSF (Figure 4C), which are well known for their potential for subsequent malignant changes [24].

#### (a) Betel nut and oral leukoplakia

OL can be defined as a predominantly white patch or plaque on the oral mucosa. Based on clinical appearance, leukoplakia can be divided into several subtypes: homogeneous (white), speckled (red/white), nodular or verrucous leukoplakia [24]. As an early sign of damage to the oral mucosa, chewers of BN or BQ with or without tobacco often develop clinically visible whitish (leukoplakia) (Figure 4A) or reddish (erythroplakia) (Figure 4B) lesions, which may or may not be accompanied by stiffening of the oral mucosa and OSF (Figure 4C). These manifestations are well-established precancerous lesions and are taken as early and important indicators of OC risk to an individual. Some 2–12% of these lesions have been reported to turn malignant over several years [3]. Although less common than leukoplakia (Figure 4A), erythroplakia (Figure 4B) poses a greater threat of cancer and lesions usually demonstrate significant epithelial dysplasia, carcinoma *in situ* or invasive squamous cell carcinoma. The presence and degree of epithelial dysplasia is generally accepted as the best indicator of malignant potential of leukoplakia, although some clinicians believe that ploidy analysis may be more reliable. There also appears to be an increased risk of transformation associated with a non-homogeneous leukoplakia, especially one that is clinically erythroplakic, verrucous or nodular [25]. One study in Taiwan indicated that the risks of developing OC after 20 years of follow-up were 42.2% for leukoplakia and 95% for erythroplakia [26]. Biopsies of leukoplakia reveal that in addition to the presence of an amorphous brown staining von Kossa positive layer on the surface, parakeratosis and atrophy of the covering oral epithelium were also observed in BN chewers. In another study, 14% of leukoplakia biopsies obtained from BN chewers demonstrated cellular atypia amounting to epithelial dysplasia [24].

It has been reported that the cessation of BN chewing resulted in resolution of 62% of leukoplakia, suggesting that BN on its own is a significant etiological factor in the development of leukoplakia. Further evidence of its relationship with BN chewing has come from the increased prevalence of this condition in subjects who suffer from OSF, which is associated strongly with the habit of BN chewing [24].

#### (b) Betel nut and oral submucous fibrosis

OSF is a chronic disorder characterized by fibrosis of the mucosa lining the upper digestive tract involving the oral cavity, oro- and hypopharynx and the upper third of the oesophagus. It was first described by Schwartz in 1952 as a fibrosing condition in five Indian women in Kenya and he called it as atrophica idiopathic atropica [27]. However, this condition is well established in medical literature since the time of *Sushrutha*, a renowned Indian physician, who lived in 2500–3000 BC and described a condition resembling OSF which he referred to as ‘*Vidari*’ [27]. There are also descriptions of similar conditions occurring in BN chewers in early texts dating back to 1908 [24]. The fibrosis is characterized by juxta-epithelial inflammatory reaction followed by chronic change in the fibro-elasticity of the lamina propria and is associated with epithelial atrophy. This leads to burning sensation in the oral cavity, blanching, and stiffening of oral mucosa and oropharynx, resulting in restricted mouth opening (Figure 4C). This condition, in turn, causes limited food consumption, difficulty in maintaining oral health, and impairs the ability to speak. The signs and symptoms depend on the evolution of lesions and number of affected sites. The malignant transformation rate of OSF has been reported to be around 7.6% over a 17-year period [24]. OSF has also been reported in several epidemiological studies mainly in the Southern states of India, among Indians living in South Africa, and among Chinese and Taiwanese [24], [27]. Other occurrences are from Pakistan, Sri Lanka, Bangladesh, Malaysia, Singapore, Thailand and Saudi Arabia with reports of sporadic incidence among Europeans [27]. OSF is also described among Asians living in Europe and the United States but who continue to chew BN [24].

It is now well accepted that all BN products, even those without tobacco, are associated with OSF, which has been established as a precancerous condition. When a paste made out of an instant BN preparation was painted into the oral cavity of albino rats, biopsies taken from the oral mucosa revealed mild to moderate loss of nuclear polarity and increase in keratoses, parakeratoses, inflammatory cell infiltration and vascularity [28]. Submucosal collagen also increased steeply and steadily throughout the study period. At the end of six months, 88.23% of biopsies showed thickened and condensed submucosal collagen, indicating submucous fibrosis [28]. It has been reported that BQ chewing with or without tobacco is a major risk factor for high prevalence of oral potentially malignant disorders (OPMD) in rural Sri Lanka [29]. The relative risk of malignant transformation in the oral mucosa of OSF patients compared to tobacco users without any precancerous lesion or condition has been estimated at around 400. Thus, BN users are potentially more liable to develop OSF and cancer over a relatively shorter duration and die earlier compared to smokers. Commercial products such as *paan masala*, *gutkha*, and *mawa* have higher concentrations of BN and appear to cause OSF more rapidly than self prepared conventional BQ, which contains smaller amounts of BN [30], [31]. Thus, the popularity of BN mixtures like *paan masala*, *gutkha* and *mawa* has spawned an epidemic of OSF, particularly among young individuals in India [32], [33].

A clear dose dependent relationship has been reported for both frequency and duration of chewing BN without tobacco in the development of OSF [34]. Only smoking and/or alcohol consumption were not found to influence the development of OSF [35], [36]. But their addition to BN chewing habit can be a risk for OSF [36].Although there is good evidence to support the role of BN as a major risk factor in the development of OSF, the mechanisms by which this occurs is not fully understood. Most studies on pathogenesis have concentrated on changes in extracellular matrix based on the premise that increased collagen synthesis or reduced collagen degradation is the possible mechanism for the development of this condition. Studies have revealed that OSF fibroblasts have marked deficiency in collagen phagocytosis, which may lead to fibrosis. In one study, arecoline was found to elevate the mRNA and protein expression of Cystatin C, a non-glycosylated basic protein consistently upregulated in a variety of fibrotic diseases, in a dose dependent manner in persons with OSF [37]. Another study showed an upregulation of Cystatin C in resident cells of buccal mucosa in OSF patients on exposure to BN. Cystatin C, in turn, inhibited the lysosomal cysteine proteases like Cathepsin B and H, resulting in decreased degradation of collagen [27]. Moreover, arecoline was also found to stimulate Cyr61 synthesis in human gingival epithelial S-G cells. Constitutive overexpression of Cyr61 protein in oral epithelial cells during BN chewing may play a role in the pathogenesis of oral cancer, since Cyr61 is associated with growth and progression of many types of tumors and is an independent poor prognostic indicator for oral cancer patients [38]. Lin *et al.* assessed the mRNA expression of histone methyltransferases, acetyltransferases, and demethylases in K-562 cells following exposure to arecoline. They observed that arecoline produced changes in the expression of several genes catalyzing histone methylation (Mll, Setdb1, and Suv39h2), acetylation (Atf2), and demethylation (JMJD6). Since H3K9 methylation is involved in maintaining the stability of heterochromatin structures and inactivating euchromatic gene expressions, this study indicates that arecoline-induced epigenetic changes play a role in the mechanisms underlying chemical-mediated cytotoxicity and genotoxicity [39].

In three separate but related studies, interleukin-6 [IL-6], keratinocyte growth factor-1 (KGF-1) and insulin-like growth factor-1 (IGF-1) expressions, which have all been implicated in tissue fibrogenesis, were significantly upregulated in persons with OSF due to BQ chewing and arecoline may be responsible for their enhanced expression [40]–[42]. Moreover, it was noticed that addition of slaked lime to BN in BQ facilitated hydrolysis of arecoline to arecaidine, which caused amplified fibroblastic proliferation and increased collagen formation. *In vitro* examination of effects of arecoline on both normal and OSF fibroblasts in culture revealed an augmented collagen synthesis by OSF fibroblasts as compared to normal fibroblasts. The reason for this elevation was thought to reflect the clonal selection of a particular cell population in the altered tissues or normal cells with somatic mutation that persists through several generations. This could be due to upregulation of pro-inflammatory and pro-fibrotic cytokines like interleukins (IL-1, IL-6, IL-8), tumor necrosis factors (TNF-α, TNF-β), platelet-derived growth factors (PDGF), fibroblast growth factors (FGF) and keratinocyte growth factor-1 (KGF-1), among others, and downregulation of interferon gamma (IFN-γ) level, resulting in fibrosis. Additionally, activation of pro-collagen genes like COL1A1, COL3A1, COL6A1 and COL7A1 has also been reported in OSF [27]. However, some studies have also shown that arecoline inhibits collagen synthesis and fibroblast proliferation *in vitro* suggesting that arecoline may have cytotoxic properties. The disparity of results from *in vitro* studies suggests that the BN may contain other agents in addition to arecoline, which are important in the pathogenesis of OSF through increased collagen synthesis [24]. It has also been reported that BQ chewing contributed to the pathogenesis of cancer and OSF by impairing T cell activation and by induction of prostaglandin E2 (PGE2), TNF-α and IL-6 production, which affect oral mucosal inflammation and growth of oral fibroblasts/oral epithelial cells [43].

Another mechanism envisions involvement of BN in the pathogenesis of OSF due to decreased collagen degradation through decreased obliteration, inhibition of phagocytosis or resistance to degradation [27]. Reduced collagenase activity and subsequently decreased degradation of collagen have been demonstrated in OSF. Polyphenols of BN, such as flavanoid, catechin and tannins cause collagen fibers to crosslink, making them less susceptible to collagenase degradation [44]. This results in increased fibrosis due to decreased collagen breakdown [45]. OSF remains active even after cessation of the chewing habit suggesting that components of the BN initiate OSF and then affect gene expression in the fibroblasts, which then produces greater amounts of collagen [46], [47]. Chewing BQ may also activate nuclear factor-kappaB (NF-κB) expression, thereby stimulating collagen synthesis by human buccal mucosal fibroblasts and leading to further fibrosis in persons with OSF [48]. In fact, OECM-1 and SAS oral keratinocytes treated with BNE activated the NF-κB pathway in a biphasic manner, particularly for SAS cells, resulting in periods of significantly elevated activity interrupted by a plateau or period of decreased activity. BNE treatment did not activate epidermal growth factor receptor signaling system, but blockage of NF-κB activation rendered the suppression of BNE-modulated COX-2 upregulation in OECM-1. Both OECM-1 and SAS oral keratinocytes also exhibited a rapid increase in c-Jun N-terminal kinases (JNK1) activity, while extracellular signal-regulated kinase (ERK) was profoundly activated in OECM-1 cells. This study thus identified that BNE induced alterations in interactive signaling systems in oral keratinocytes could be a basis of the pathogenicity of BN [49]. Additionally, reduced level of main gelatinolytic proteinases secreted by buccal mucosal fibroblasts (BMF), namely matrix metalloproteinases MMP2, MMP9 and elevated levels of tissue inhibitor of metalloproteinase-1 (TIMP-1) have been reported in OSF as a possible means of loss of equilibrium of extracellular matrix (ECM) in OSF. This may result in increased and continuous deposition of ECM. In fact, arecoline and safrole significantly elevated TIMP-1 protein and mRNA expression in BMF, and this is a possible pathogenesis for OSF [50]. In contrast, MMP-2 and MMP-9 have been reported to be present in human OSCC and the activated MMP-2 could be the main enzyme for gelatinolysis in OSCC, facilitating invasion and metastasis [51]. One study assessed the change in salivary MMP-9 protein levels 2 hours after 5-minute BQ chewing stimulation (BQCS) in non-BQ users and the expression profile of this proteinase in saliva and tumor specimens of OSCC patients with a history of BQ use. MMP-9 was found to be upregulated in response to BQCS and MMP-9 expression was also associated with neck lymph node metastasis, thus implying a significant role of MMP-9 in the progression of OSCC among patients with a history of BQ use in Taiwan [52].

Raised copper concentrations have been shown in products containing BN in comparison to other nut based snacks. It has also been observed that chewing BN for 5–30 min significantly raised the soluble Cu level in saliva. Study of buccal mucosal biopsies from patients with OSF indicated raised Cu level [53]. Addition of CuCl_2_ increased the collagen synthesis by the oral fibroblasts. However, the addition of CuCl_2_ neither increased the synthesis of non-collagenous proteins by the fibroblasts nor influenced their proliferation rate. These *in vitro* results support the hypothesis that Cu in BN acts as a mediator of OSF [54]. This has led to the hypothesis that the increased tissue Cu may increase the activity of the enzyme lysyl oxidase, which is a Cu-dependent enzyme that has been implicated in the pathogenesis of several fibrotic disorders, including OSF [24]. Cu salts significantly increased the production of collagen by oral fibroblasts *in vitro* supposedly by upregulation of activity of a Cu-dependent enzyme, lysyl oxidase, which catalyses the cross linking of collagens and elastin [6]. The collagen cross linked with lysyl oxidase is rendered insoluble and is shown to be ten times more resistant to digestion by mammalian collagenase [27]. Further, a significant gradual increase in serum Cu levels from pre-cancer to advanced cancer in patients has been documented [55], which may have a role in oral fibrosis to cancer pathogenesis (Figure 3).

### 6. LINK BETWEEN BETEL NUT AND CARCINOGENESIS

There exists an accumulated wealth of historical evidences suggesting that the BN may be involved in the development of OSCC (Figure 5C) [24]. Recent research has also generated sufficient evidence to implicate BN as well as BQ, with or without tobacco, as a suspected carcinogen to humans [4], [5], [9]. In addition to OC, significant increase in the incidence of cancers of the esophagus, liver, pancreas, larynx and lung were seen among BN chewers [56]. A study of esophageal squamous cell carcinoma (ESCC) in Taiwan revealed that subjects who chewed between 1 and 495 BN or more than 495 BN per year had 3.6-fold and 9.2-fold higher risk, respectively, of developing esophageal cancer compared to those who did not chew BN [57].

A causal association between tobacco and BQ chewing habits and oral mucosal diseases such as OL, OSF and OC has been established and heavy users have a significantly increased mortality rate [6]. A study in Taiwan has suggested that elimination of BN may prevent 62% of OL and 26% of malignant transformation to OC in the underlying population [58]. Data on oral cavity cancer in Taiwan from the period between 1986 and 1997 also indicated that those who chewed BN had a higher risk for OC [59]. Studies in OC patients demonstrated that male cases were far more common than females, comprising 90–93% men and 7–10% women. It is proposed that this gender difference may be explained by the prevalence of lower proportion of BQ chewing habits among females. Concerns about the disfiguring effects of BQ chewing, including red staining of lips and teeth, and foul smelling breath, are frequently reported by females, which may account for gender differences in head & neck cancer (HNC) prevalence among females and males [60]. Chewing BQ independently was found to contribute to the risk of HNC and the estimated prevalence of BQ chewing in Taiwanese patients with HNC was found to be approximately 85% [60]. Chewing BQ without tobacco has also been implicated in the causation of OC among South Asian communities [61].

A study of OSCC and concomitant oral habits undertaken in South African Indians revealed that 68% women with cheek cancer and 84% with tongue cancer only chewed BN without tobacco, snuff or smoking. The data show that the BN habit with or without tobacco is important in the development of OSCC and it has been suggested that elimination of this habit can reduce the risk in these women substantially (89–91%) if all other factors remained the same [46]. Time-trend analysis of cancers at all sites for the period 1990–1996 showed a decrease in cancers of the oral cavity in Indian population based registries, but an increase in the incidence of OC was reported among those aged <50 years between 1983–1987 and 1995, consistent with the hypothesis of an increase in OC among the young due to increased consumption of the alternative chewing products such as, *gutkha* and *paan masala* [6]. BN without tobacco was, thus, recognized as a group I carcinogen to human by the International Agency for Research on Cancer (IARC) and World Health Organization (WHO) in 2004 [9]. In addition to human data, there are also a large number of experimental studies, which have reported the carcinogenic potential of BN and its derivatives both *in vivo* and *in vitro* [24].

#### (a) *In vivo* studies

Several animal studies have confirmed that BN products and derivatives, such as arecoline and BN derived nitrosamines, also referred to as betel specific nitrosamines (BSNA), have the ability to induce neoplastic changes in experimental animals (Figure 3). Alkaloids of BN are suspected to be its main carcinogenic constituent [2]–[6], [9], [24], [62]. Early studies found that the application of arecaidine to the oral mucosa of experimental animals failed to have any carcinogenic effects unless it was supplemented with a known promoter, such as croton oil [24]. However, arecoline administered by gavage produced lung adenocarcinoma, stomach SCC and liver haemangioma in male mice [9]. Cheek pouch application of arecoline following application of slaked lime produced an esophageal papilloma in female hamsters, while local application of arecaidine to the cheek pouch did not produce tumor in male hamsters [9]. To explain the variable observation, it is proposed that the alkaloids first required metabolic activation via nitrosation to develop its carcinogenicity [63]. *In vitro* data suggest that arecoline is metabolized by carboxylesterase in mouse liver and kidney. Male Swiss albino mice fed BN powder or arecoline showed enhanced levels of the hepatic cytochrome P450 and b5 and decreased levels of hepatic GSH [9]. In fact, human cytochrome P450 was found to be involved in the mutagenic activation of BSNA such as 3-(N-nitrosomethylamino)propionitrile (NMPN), 3-(N-nitrosomethylamino)propionaldehyde (NMPA) and N-nitrosoguvacoline (NG) using genetically engineered *Salmonella typhimurium* YG7108 expressing each form of human P450 together with NADPH-P450 reductase [64]. Exposure of Swiss albino mice to arecoline was found to lower poly-ADP-ribosylation (PAR) of most cellular and histone proteins and induce relaxation of chromatin, thereby allowing the N-nitrosamines of arecoline easy access to genomic DNA for interaction, while the absence of PAR mediated repair may favour the accumulation of DNA damage [65]. Arecoline induced micronuclei (MN), chromosomal aberrations (CA) and sister chromatid exchange (SCE) in bone marrow cells (BMC) of Swiss albino mice [66], [67]. The arecoline induced DNA damage was found to be influenced by endogenous GSH levels with the frequency of CA and SCE increasing when arecoline was given to mice treated with buthioninesulfoximine (BSO), a GSH synthesis inhibitor (Figure 3).

The frequency of SCE was found to be elevated in mouse BMC when mice were exposed to the aqueous extract of betel nut (AEBN) and its tannin [67]. AEBN also induced micronucleated cells (MNC) in BMC of Swiss albino mice [5]. Hamsters fed with powdered diet containing BN or BQ showed significant decrease in the survival rate, body weight, and hyperkeratosis and acanthosis of cheek pouch indicating that BN and BQ components may induce alterations in proliferation and differentiation of oral epithelial cells [68]. When the buccal mucosa of mice was treated regularly with a topical application of water based BNE, the oral epithelium showed progressive changes in epithelial thickness leading to atrophy, increased cellularity of fibroblasts, fibrosis of connective tissue, focal infiltration of inflammatory cells and muscle atrophy [69]. Frequency of all the three cytogenetic endpoints, viz. CA, SCE and MNC, were found to be elevated significantly in a dose dependent manner in cultures exposed to aqueous extracts of *paan masala* without metabolic activation [70]. The carcinogenic and tumor promoting potentials of an ethanolic *paan masala* extract (EPME) were determined using the hairless skin of S/RVCri-ba or Bare mice and the forestomach and esophagus of ICRC mice as the target tissues. EPME promoted skin papilloma formation and enhanced the rate of conversion of papilloma to carcinoma. Induction of mild epidermal hyperplasia, dermal edema, increase in epidermal mitotic activity and the rate of epidermal and dermal DNA syntheses by EPME correlated well with its skin tumor promoting potential. In ICRC mice, EPME was inactive as a complete carcinogen, but effectively promoted the development of forestomach and esophageal papilloma and carcinoma in a concentration dependent manner indicating that habitual *paan masala* use may exert carcinogenic and co-carcinogenic influences [71]. Exposure of male and female mice to *paan masala* revealed a significant dose dependent increase in lung adenocarcinoma but not in liver and stomach [72].

#### (b) *In vitro* studies

BNE was found to decrease cell survival, vital dye accumulation and membrane integrity of cultured human buccal epithelial cells in a dose dependent manner. BNE also caused formation of both DNA single strand breaks and DNA protein cross links [63], [73], [74]. Different BNE, such as aqueous extract of betel nut (AEBN), acetic acid extract of betel nut (AAEBN), HCl extract of betel nut (HEBN) and ethanol extract of betel nut (EEBN) as well as arecoline showed different extents of cytostatic and cytotoxic effects, and induced variable levels of dose dependent unscheduled DNA synthesis (UDS) in Hep2 cells *in vitro*. In manifestation of these effects arecoline, HEBN and EEBN were most potent [73], [75]. Cultured normal human oral keratinocytes (NHOK) exposed to ripe BNE also showed significant decrease in population doubling, increase in senescence, cell cycle arrest at G1/S phase and decrease in cell proliferation [76]. It has been reported that BQ may accelerate tumor migration by stimulating MMP-8 expression through MEK pathway in at least some carcinomas of the upper aerodigestive tract. Furthermore, arecoline may be one of the positive MMP-8 regulators among BQ ingredients [77]. Investigation of prostaglandin endoperoxide synthase (PHS) action on the growth of OC in response to BNE exposure of two human oral carcinoma cell lines OEC-M1, and KB, and one normal fibroblast cell line, NF, revealed that BNE significantly inhibited the cell growth of OEC-M1, KB and NF. PHS activity in OEC-M1 and NF was significantly increased by low BNE concentrations but significantly reduced at higher concentrations. The PHS activity in KB, on the other hand, was significantly inhibited by BNE and this effect was intensified as concentration increased [78]. Treatment of human oral mucosal fibroblasts (OMF) with BNE or arecoline induced about 3-fold increase in mRNA levels of the proto-oncogene c-*jun* independent of GSH depletion [79].

The BNE and inflorescence of *Piper betle* (IPB) also induced DNA strand breaks. In addition, BNE, IPB, the BN polyphenol, catechin as well as arecoline decreased cell survival and proliferation. In contrast, another component of BQ, the aqueous extract of lime, was found to increase cell proliferation [80]. AEBN was found to reduce endogenous glutathione (GSH) level, induce CA and delay cell kinetics in mouse BMC with the induction of SCE probably involving TP53 dependent changes in cell proliferation [81]. Ethyl acetate and *n*-butanol extracts of BN as well as betel leaf are reported to induce CA in human lymphocytes and Chinese hamster ovary (CHO) cells [4]. All components of BQ have been shown to individually enhance chromatid breaks and exchanges in the range of 12–37% in human cells *in vitro*. AEBN also induced DNA strand breaks and enhanced cell proliferation in mouse kidney T1 cells *in vitro* [73]. BNE exposure to CHO-K1 cells caused increased MN frequency, G2/M arrest, cytokinesis failure and an accumulation of hyperploid/aneuploid cells. These events are associated with an increase in intracellular H_2_O_2_ level and actin filament disorganization [82]. BNE also elicited actin reorganization resulting in fibroblastoid morphological change, genesis of lamellipodia, loss of subcortical actin and stress fiber formation in cultivated NHOK cells [83].

Arecoline alone has been reported to inhibit cell attachment, cell spreading and cell migration in a dose dependent manner in cultured human gingival fibroblasts (HGF) [84]. In fact, GSH depletion and reduction of glutathione *S*-transferase (GST) activity have been demonstrated in cultured human oral keratinocytes and in fibroblasts treated with arecoline [9]. Arecoline was also reported to be cytotoxic to human buccal fibroblasts in a dose dependent manner wherein the cellular GST activity was downregulated in a dose dependent manner without increase in lipid peroxidation. Addition of extracellular nicotine acted synergistically on the arecoline induced cytotoxicity, indicating that arecoline may render human OMF more vulnerable to other reactive agents in cigarettes via GST reduction. These observations could explain why patients who practice the combined habit of BQ chewing and cigarette smoking are at greater risk of contracting OC [85]. Arecoline inhibited growth of human KB epithelial cells in dose- and time-dependent manners by causing cell cycle arrest in late S and G2/M phases due to induction of cyclin Bl, Wee 1, and phosphorylated cdc2 proteins and inhibition of p21 protein expression in KB cancer cells. In primary human gingival keratinocyte (HGK) cell line, arecoline effect appeared to be mediated differently. In this case, arecoline induced p21 but inhibited cdc2 and cyclin B1 proteins. This clearly suggests that differential regulation of S and/or G2/M cell cycle related proteins in the HGK and KB cells play crucial roles in different stages of BQ mediated carcinogenesis [86]. Arecoline could also induce γ-H2AX phosphorylation, a sensitive DNA damage marker, in KB, HEP-2, and 293 cells, suggesting that DNA damage was elicited by arecoline. Moreover, the expression of p53 regulated p21 (WAF1) and p53 activated DNA repair were repressed by arecoline [87]. Arecoline was cytotoxic to HGF cells due to depletion of intracellular thiols and inhibition of mitochondrial activity and induced cell cycle arrest in HGF cells at G2/M phase in a dose dependent manner [88]. Global gene expression profiling in HGF exposed to arecoline revealed that four genes related to maintenance of genome stability and DNA repair were repressed by arecoline [89]. They are *FANCG*, also known as *XRCC*9 (tumor suppressor capable of correcting CA), *CHAF*1 and *CHAF*2 (encoding chromatin assembly factor I or CAF1) and *BRCA*1 (breast cancer susceptibility gene implicated in DNA damage response and DNA repair). Among them, at least the *BRCA*1 response was dose dependent. *COX*-2 and *PTGS*2, which are involved in cancer initiation and progression, were over expressed in HGF cells. *HSP*4*A*1 and *DNAAJA*1, which belong to the *HSP*70 family of stress-induced proteins and GDF15/MIC-1, were also upregulated by arecoline in dose dependent manner [89]. Chen *et al.* established two oral cancer sublines chronically treated with BNE and used methods such as microarray and immunohistochemistry to screen and validate the genes exhibiting altered expressions in BNE sublines or in cancer tissues. They found that a total of 35 genes were differentially expressed in both sublines. Several functional pathways were significantly altered. Six genes were confirmed over 2-fold of changes, including *Ches1*. Functional analyses showed that overexpression of Ches1 suppressed cell growth and arrested cells in the G2/M phase. They thus concluded that loss of *Ches1* may be attributed to BNE-induced oral carcinogenesis [90].

Treatment of normal human oral fibroblasts with BNE was also reported to alter miRNA expression profile. BNE-induced overexpression of miR-23a was found to be correlated with an increase of γ-H2AX, a DNA damage marker. *FANCG* was confirmed to be a target of miR-23a by ectopic overexpression or knockdown of miR-23a. The correlation between miR-23a overexpression and BN-chewing habit was also reported in oral cancer patients. Thus, BNE-induced miR-23a was correlated with a reduced *FANCG* expression and DNA double strand break (DSB) repair, which might contribute to BNE-associated human malignancies [91]. Oral fibroblasts with chronic subtoxic BNE treatment were found to exhibit growth arrest and MMP-2 activation. The supernatant of these arrested oral fibroblasts activated the AKT signaling pathway in oral carcinoma cells. Moreover, subcutaneous co-injection of arrested oral fibroblasts into nude mice significantly enhanced the tumorigenicity of xenographic oral carcinoma cells. The investigators therefore concluded that BNE may impair oral fibroblasts and then modulate the progression of oral epithelial oncogenesis *via* their secreted molecules [92].Various studies have clearly established the mutagenecity of BN and its components. The major metabolite of arecoline, arecoline N-oxide, is reported to be moderately mutagenic to *Salmonella typhimurium* tester strains TA 100 and TA 98. But this mutagenicity was potently inhibited by glutathione, N-acetylcysteine, and cysteine [93]. Aqueous extracts of BQ without tobacco induced mutations in *Salmonella typhimurium* but not in Chinese hamster V79 cells. AEBN, on the other hand, induced mutations in *Salmonella typhimurium* and in Chinese hamster V79 cells besides inducing gene conversion in *Saccharomyces cerevisiae* as well as CA in CHO cells. BN tannin fraction induced gene conversion in *Saccharomyces cerevisiae* [4]. Ames test using *Salmonella typhimurium* strain TA 1535 revealed that arecoline, AEBN and HEBN were weak mutagens while AAEBN and EEBN were strong mutagens suggesting that the mutagenic potential of arecoline could be significantly enhanced by other constituents of BN [5], [94], [95]. Exposure to BNE was also found to induce mutation at the *hypoxanthine phosphoribosyltransferase* (HPRT) locus in human keratinocytes, which also increased frequency of appearance of MN, intracellular levels of reactive oxygen species (ROS) and 8-hydroxyguanosine in the cells suggesting that stress caused by long term BNE exposure enhanced oxidative stress and genetic damage in human keratinocytes [96].

#### (c) Human studies

Among BN chewers, the possible genomic damage caused by BN without tobacco was confirmed in cytogenetic studies. BNE has been shown to be cytotoxic and genotoxic to human buccal epithelial cells [74]. This may be correlated to its ability to increase DNA strand breaks, MNC formation, gene mutation and CA [16], [67]. A study aimed to evaluate the genotoxic effect of BN and tobacco on human peripheral blood lymphocytes revealed anomalies. Binucleated cells with MN, total MN, nucleoplasmic bridge and nuclear buds were higher in chewers whereas elevation in binucleated MN and total MN were significant among subjects with oral submucous fibrosis than nonchewers. Significant positive correlation was also observed between induction of cytokinesis-blocked micronucleus (CBMN) and consumption of BQ per day [97]. However, there is still a void in the complete understanding of the molecular mechanism by which BN affects DNA repair and genome stability genes. These two are hallmarks of genome fidelity. Arecoline inhibited both expression and transactivation functions of p53. This inhibition is proposed to play an important role in arecoline mediated suppression of DNA repair. It was shown that the expression of p53 mRNA was frequently downregulated in BQ associated OC [87]. Arecoline also arrested cells at prometaphase with large amounts of misaligned chromosomes by stabilizing mitotic spindle assembly, which led to distorted organization of mitotic spindles, misalignment of chromosomes and upregulation of spindle assembly checkpoint (SAC) genes [98]. A chromosomal analysis of patients with OC primarily associated with BN consumption using comparative genomic hybridization revealed that the most common gains of chromosome arms were 8q, 9q and 11q, and the most frequent losses were of chromosomal arms 3p and 4q [99].

A study revealed that OSF was largely associated with BN and the exfoliated oral mucosal cells of such patients had significantly higher numbers of MNC. The patients also exhibited increased SCE in circulating blood lymphocytes indicating that the carcinogenic agents in BN produce damage not only in target tissue but also in other tissues [100]. Rooban reviewed the effects of different ways of taking arecoline on salivary flow rates (SRF) and pH of saliva [101]. With an increase in frequency and exposure time of chewing raw BN, both SFR and pH increased. In processed BN chewers, increase in duration and frequency of consumption increased the SFR and decreased the pH, respectively. For chewers taking BN with tobacco, increase in duration was significantly associated with decrease in salivary pH. Similarly, IBP, which contains safrole (4-allyl-1,2-methylenedioxybenzene), a unique ingredient of BQ in Taiwan, forms Safrole–DNA adducts. This has been suggested to play an important role in OC in the population of Taiwan. A high frequency of safrole-like DNA adducts has been reported in BQ associated OSCC and noncancerous matched tissue in contrast to the absence of such adducts in all of non-BQ associated OC. Safrole-DNA adducts are present in oral cancer tissue from patients who have chewed BQ containing high concentration of safrole as well as in peripheral white blood cells. Safrole is classified as a rodent hepatocarcinogen, and chewing BQ may contribute to human exposure to this compound. The saliva of a person chewing BQ contains on average 420 µmol/L of safrole. Interestingly, safrole-DNA adducts were found in liver biopsy specimens of a Taiwanese man suffering from hepatocellular carcinoma, who had chewed BQ for over 32 years. This implies that safrole may be implicated not only in carcinogenesis of the oral cavity of BQ chewers through direct contact, but can also be transported via the oral-digestive tract to distant organs like the liver where it acts a likely cause of liver carcinogenesis [102]. Moreover, individuals with at least one cytochrome P450 - CYP2E1c2 allele had a significantly higher frequency of safrole-DNA adducts formation than those with the CYP2E1c1c1 genotype while chewing less than 20 BQ per day [103]. Hydroxychavicol, a phenolic component of betel leaf, has been found in human saliva at a 4.6 mM concentration after BQ chewing. Hydroxychavicol may induce DNA single strand breaks and 8-hydroxydeoxyguanosine, a marker of oxidative DNA damage, in cultured cells [104].

Hung *et al.* reported the upregulation of Asb6, a coupling protein to the APS adapter protein, which is involved in insulin signaling for glucose transportation, of normal keratinocytes and oral cancer cells under BNE treatment. They also demonstrated a positive correlation between Asb6 upregulation (cancerous tissues versus adjacent normal tissues) and clinicopathological features such as poor survival status in OSCC patients [105].

In a study pertaining to the contribution of combined BN chewing and cigarette smoking to the risk of OSCC in Taiwan, Wu *et al.* revealed that the alkaline environment created in the oral cavity of BN chewers by lime may enhance nicotine-related oral carcinogenesis through a synergistic effect of nicotine and the alkalinity in inducing higher expression of phosphorylated AKT [106]. AKT/protein kinase B (PKB) is a serine/threonine kinase which is implicated in mediating a variety of biological responses including cell growth, proliferation and survival. AKT is activated by phosphorylation on two critical residues, namely threonine 308 (Thr308) and serine 473 (Ser473), and several studies have found AKT2 to be amplified or overexpressed at the mRNA level in a number of human malignancies [107].

A study involving patients of HNC suggested that BQ chewing may increase mitochondrial DNA (mtDNA) mutation in human oral tissues and that accumulation of mtDNA deletions and subsequent cytoplasmic segregation of these mutations during cell division could be important contributors to the early phase of OC [108]. ZASC1, a zinc finger transcription factor localized on 3q26, is frequently amplified in OSCC. Examination of OSCC patients revealed that increase of ZASC1 gene copy number in recurrent tumors was associated with the consumption of BQ in patients [109]. O(6)-methylguanine-DNA methyltransferase (MGMT) ameliorates mutagenic, carcinogenic and cytotoxic adducts from O(6)-methylguanine in DNA. The absence of MGMT expression associated with promoter hypermethylation has been reported to be related to BQ chewing and, thus, might be a significant event in OC [110]. A high frequency of hypermethylation of p14, p15 and p16 was also detected in the precancerous lesions of BQ chewers in Sri Lanka [111]. Further, it has been proposed that epigenetic silencing of RASSF1A and p16INK4a gene expressions by promoter hypermethylation may play critical roles in BN associated OC [112].

Alphavbeta6 (αvβ6) integrin is capable of promoting both tissue fibrosis and carcinoma invasion, and has been reported to be markedly up-regulated in OSF [113]. Moutasim *et al.* investigated the functional role of αvβ6 using oral keratinocyte-derived cells genetically modified to express high αvβ6 (VB6), and also NTERT-immortalized oral keratinocytes, which express low αvβ6 (OKF6/TERT-1). VB6 cells showed significant αvβ6-dependent activation of TGF-β1, which induced transdifferentiation of oral fibroblasts into myofibroblasts and resulted in up-regulation of genes associated with tissue fibrosis. These experimental *in vitro* findings were confirmed using human clinical samples, which revealed that the stroma of OSF contained myofibroblasts and that TGF-β1-dependent Smad signaling was detectable both in keratinocytes and in myofibroblasts. The investigators also found that arecoline, the major alkaloid of BN up-regulated keratinocyte αvβ6 expression. This was modulated through the M_4_ muscarinic acetylcholine receptor and was suppressed by the M_4_ antagonist, tropicamide. Arecoline-dependent αvβ6 up-regulation promoted keratinocyte migration and induced invasion, raising the possibility that this mechanism may support malignant transformation. This study thus suggests that the pathogenesis of OSF may be epithelial-driven and involve arecoline-dependent up-regulation of αvβ6 integrin [113].

Heat shock protein 47 (HSP47) is a product of CBP2 gene located at chromosome 11q13.5, a region frequently amplified in human cancers. HSP47 expression was reported to be significantly higher in OSCC specimens than normal epithelium, while lower HSP47 expression was associated with lymph node metastasis. No significant difference in HSP47 expression was observed with respect to age, sex, tumor category, tumor stage and differentiation. Furthermore, arecoline was found to elevate HSP47 expression in a dose- and time-dependent manner in oral epithelial cell line OC2. This study therefore concluded that HSP47 could be used clinically as a marker for lymph node metastasis of oral carcinogenesis [114].

### 7. BETEL NUT AND TUMOR SUPPRESSOR GENES

The *p53* gene is known to be mutated in a variety of human and experimental animal cancers. Similarly, change in cellular level of p53 protein is also known to occur. Accumulation of p53 protein or its stabilization is an important indicator of the presence of mutant p53 protein [115], [116]. However, reports pertaining to *p53* mutation status of cancers associated with BN chewing have been widely contradictory. Exposure to BN and/or BQ with or without tobacco has been reported to result in high incidence of *p53* mutations in Taiwanese, Thai, Sri-Lankan and North Indian Population; however, similar exposures resulted in low frequency of *p53* mutations in the populations of India and Papua New Guinea, in other reports. Despite these conflicting observations, a common trend observed among the varying populations was p53 overexpression and nuclear accumulation of p53 protein (see Table 1) [117]–[127]. A study on northeastern Indian population also found significant influence of p53 codon 72 polymorphism, with interaction between p53 genotype and smoking resulting in a significant risk of OC, while interaction of p53 genotypes with BQ leading to a significant risk of lung cancer [128]. Another study on cancer patients in northeastern India also found that the subjects with family history of cancer were more likely to develop ESCC if they were BQ users and germ line mutations in the DNA repair gene, BRCA2, played a role in this familial aggregation of ESCC [129].

**Table 1:** p53 associated alterations in betel nut (BN) and/or betel quid (BQ) associated human precancerous lesions/cancers.

#	Exposure condition	Effect(s)	P53 associated alteration(s)	Reference
1	BQ and alcohol	ESCC in Taiwanese population	A:T**→**G:C transition and G:C**→**T:A transversion	117
2	BQ	Atrophic oral lichen planus (OLP) in Taiwanese patients	Higher expressions of p53 and proliferating cell nuclear antigen (PCNA)	118
3	BN	Oral cancer in Thailand	Mutations detected in 11.8% (8/68) of betel-related tumors and 7 of 8 mutations were G:C to A:T transitions	119
4	BQ	Leukoplakia and OSCC in North Indian population	p53 missense mutations, p53 antibodies and p53 protein accumulation	120
5	BQ and tobacco	OSCC in Taiwanese population	G:C**→**A:T transitions	121
6	BQ and tobacco	OSCC in Indian population	Low incidence of p53 mutations	122
7	BQ	OSCC in Sri Lankan population	Point, small deletion and addition type of mutations mainly clustered in exon 5 of the *p53* gene	123
8	BQ and tobacco	OSCC in Taiwanese population	Mutations in codons 273–282 in exon 8 of p53, nuclear accumulation and positive p53 immunostaining	124
9	BN and tobacco	OSCC in South Indian population	Nuclear p53 staining and p53 expression	125
10	BQ without tobacco	Oral cancers from Papua New Guinea	Low frequency of p53 mutations	126
11	BN	OSCC in Sri Lankan population	Over expressed p53	127
12	BQ and tobacco	Lung cancer and oral cancer in North east Indian population	P53 codon 72 polymorphism	128

In previously reported transgenerational studies, 6 week old male and female Swiss albino mice were exposed chronically to AEBN in drinking water at a dose of 2 mg ml^−1^ for 24 weeks. These mice are referred to as the chronically exposed P generation mice. The F1 generation was raised by inbreeding of P generation mice exposed to AEBN for 6 weeks. Similarly, the F2 and F3 generations were raised from AEBN exposed F1 and F2 mice, respectively [130], [131]. Thus, the transgenerationally exposed F1, F2 and F3 mice received low dose AEBN prior to and during conception and the entire period of development and maturation. In these studies, exposure to AEBN was found to severely impair the ultrastructure of the nucleus, endoplasmic reticulum and mitochondria with a significant reduction in mitochondrial index from the P through F3 generations. This indicates a progressive loss of apoptosis with progression of generation [132]. It was further observed that the exposure to AEBN resulted in an immediate upregulation of p53 protein up to 2–2.5 folds after 6–8 weeks, and Brca1 and Brca2 proteins to 1.4 folds after 2 weeks of exposure. Subsequently, the p53 protein declined to control level and the Brca1 and Brca2 proteins to 70% of the control after 16 weeks of exposure concomitant with the appearance of preneoplastic nodules in the liver. In contrast, in the transgenerationally exposed mice, the level of p53 protein remained largely invariant, and the levels of Brca1 and Brca2 proteins declined rapidly below control level without recording an initial increase. The appearance of pre-neoplastic nodules of the liver was significantly advanced in the transgenerationally exposed mice; developing in 8 weeks in F1, 6 weeks in F2 and 4 weeks in F3 mice. This clearly exhibits progressively increasing genomic instability due to prolonged AEBN exposure and enhanced cancer predisposition.

DNA sequence analyses revealed no mutation in exons 5 and 7 of the *p53* gene and the amplified segment (nucleotides 1–257) of exon 27 of the *Brca2* gene in P, F1, F2 and F3 mice. In contrast, a mis-sense mutation (G→C) was observed in exon 11 of the *Brca1* gene in F1, F2 and F3 mice, but not in P mice. Such a mutation would result in corresponding amino acid replacement Cys→Ser. *In silico* protein modeling revealed that the amino acid substitution was likely to cause structural alterations in the RAD50 binding region of the Brca1 protein, which is crucial for its function in error free repair of DNA single and double strand breaks. These observations clearly indicate that the *p53, Brca1* and *Brca2* tumor suppressor genes are intrinsically involved in the process of BN induced carcinogenesis in mice as well as in the transgenerational transmission of carcinogenic risk following AEBN exposure. The p53, Brca1 and Brca2 responses were abrogated in the mice exposed transgenerationally to AEBN resulting in significantly increased predisposition to cancer [130], [131]. The inactivation of the *p53* gene, which apparently played a crucial role in BN associated cancer in mice, was not achieved through *p53* mutation. The mechanism of p53 inactivation may also involve other routes, which requires to be investigated in the future. One possible alternative mechanism for p53 inactivation in BN induced carcinogenesis may be over expression of MDM2 protein, which has been shown in OSCC [133]. A high prevalence of MDM2 protein was also found in BQ chewing associated OSCC in Taiwan [134]. MDM2 protein has been shown to negatively regulate the function of p53 tumor suppressor protein through two main mechanisms. First, the direct binding of MDM2 to the N-terminal end of p53 inhibits the transcriptional activation function of p53. Second, MDM2 possesses E3 ubiquitin ligase activity that targets p53 for modification and subsequent degradation through the 26S proteasome [135]. Overexpression of MDM2 would therefore lead to carcinogenesis in a p53-dependent manner.

### 8. BETEL NUT POLYPHENOLS AND TANNINS IN CARCINOGENESIS

Toxicity studies relating to BN specific polyphenols and tannins are not conclusive with both carcinogenic and anti-carcinogenic effects being reported in literature. It is reported that ROS produced during autooxidation of BN polyphenols in the BQ chewer's saliva might be crucial in the initiation and promotion of OC [63]. However, the polyphenols are primarily known to be strong antioxidants and, thereby, also considered a food supplement that reduces the risk of degenerative diseases. Huang *et al.* have reported that the antioxidant capacity of the BNE procyanidins increased with the degree of polymerization. Further, they have also demonstrated that BNE which contains catechins based oligomeric and polymeric procyanidins, regulates COX-2 expression *in vitro* and possess anti-inflammatory potential *in vivo* [14]. Similarly, incidence of certain cancers, such as esophageal cancer, has been reported to correlate well with the consumption of tannins-rich food, such as BN, suggesting that tannins might also be carcinogenic. However, other reports indicated that the carcinogenic activity of tannins might be related to components associated with tannins such as “flavolans”- the polymers formed by condensation of flavans, referred to as polyflavonoid tannins or condensed tannins, rather than tannins themselves [136]. More research is required to properly understand this aspect.

### 9. BETEL NUT AND HUMAN GENETIC SUSCEPTIBILITY TO ORAL CANCER

Exposure to BN derived carcinogens, particularly alkaloids, enhance the risk of cancer in BN or BQ chewers in general. However, correlation between prevalence of cancer in human populations in different parts of the world and habit of BN/BQ mastication is not absolute. This suggests that the genetic makeup of the masticator has its own influence on the ultimate manifestation of BN induced cancer. It is becoming obvious that the interplay between the genetic constitution and the environmental factor(s), determine the final risk of human cancers, especially OC, following the exposure to BN or BQ alone or in combination with additives, including tobacco. Mere exposure to BN or BQ may not commit the chewer to cancer. For any given level of exposure to BN carcinogens, only a proportion of exposed individuals will develop cancer, indicating the prevalence of inter-individual differences in susceptibility [137]. Individual susceptibility to cancer may originate from several factors, including (a) differences in metabolism influencing the metabolic activation of BN derived carcinogens, (b) status of DNA repair pathways and related genes, (c) patterns of expression of proto-oncogenes and tumor suppressor genes and (d) nutritional status of the masticator, etc. Variations in an individual's metabolic phenotype, i.e., phenotypic polymorphism, have also been detected in a variety of enzymes involved in activation and detoxification of chemical carcinogens. It is becoming clearer now that different phenotypic and/or metabolic variations stem from genetic polymorphisms prevalent in different population groups [138]. A number of genetic polymorphisms have been identified, which seem to be associated with the risk of BN induced preneoplastic lesions or pre-cancers, like OL/OE and OSF, as well as with the development of OC in human subpopulations in different regions of the world. These polymorphisms have been mapped to genes with diverse function. However, polymorphisms in a few genes appear to be more significant; these include the DNA repair genes, *XRCC4* in Taiwanese population and *XRCC1* and *XPD* in Indian population, genes encoding detoxifying enzymes such as *NAT2* encoding the most important phase II metabolic enzyme for BQ in Taiwanese population, *GSTT1* and *GSTM1* in Indian and Thai populations, and *CYP2A6* in Sri-Lankan population and genes encoding matrix metalloproteinases, such as *MMP9* in Taiwanese population and *MMP3* in Asian population (see Table 2) [139]–[165]. This clearly indicates the extremely complex and highly variable influence of the genetic makeup of the population groups on their cancer susceptibility. Further research in this area is also warranted.

**Table 2:** Genetic polymorphisms and their role(s) in betel nut (BN) and/or betel quid (BQ) associated human precancerous lesions/cancers.

#	Gene/region	Genetic polymorphism(s)	Effect(s)	Reference
1	Nuclear factor-kappa B (NF-κB)	Genetic polymorphisms of *NFKB1* and *NFKBIA*	*NFKB1* −94 ATTG2, *NFKBIA* −826 T and −881 G alleles are associated with oral carcinogenesis. The combination of *NFKB1* or *NFKBIA* gene polymorphisms and tobacco and betel consumption appears related to an increased risk of oral cancer. The genetic polymorphism of *NFKBIA* −519 might be a predictive factor for the distal metastasis of OSCC in Taiwanese	139
2	Survivin gene	Genetic polymorphisms of survivin gene	The survivin −31GG, +9194 GG, and +9809 TT homozygotes exhibited higher risk for oral cancer compared with the corresponding ancestral genotype, and +9809 SNPs combined with betel quid chewing and/or tobacco consumption could robustly elevate susceptibility to oral cancer. The distribution frequency of the −31 G: +9194 A: +9809 T haplotype was significantly higher in oral cancer patients than in control participants, in Taiwanese men	140
3	Epidermal growth factor receptor (EGFR) gene	Q787Q silent mutation	A high frequency of Q787Q mutation in BN chewing associated Taiwanese OSCC patients	141
4	Chemokine (C-C motif) receptor 2 gene CCR2	V64I CCR2 gene polymorphism	Individuals with GA or at least one A allele had a higher risk for oral cancer, compared to GG genotypes. Moreover, for subjects with GA or at least one A allele of V64I CCR2 gene polymorphism, those exposed to environmental risk factors including alcohol, tobacco and *Areca* consumptions possessed a significantly higher risk for oral cancer than those unexposed subjects in Chinese population	142
5	Cytochrome gene, *CYP26B1*	Genetic polymorphism of CYP26B1	Genetic polymorphism AA of CYP26B1 appeared to correlate with the risk of oral squamous cell carcinoma (OSCC), and chewing BQ multiplicatively interacted with CYP26B1 AA to increase the OSCC risk in Taiwanese population	143
6	Urokinase plasminogen activator (uPA) gene and plasminogen activator inhibitor (PAI)-1	Genetic polymorphisms of uPA gene. At least one 5G allele or 4G/4G genotype of PAI-1	Combination of uPA system gene polymorphisms and betel nut and tobacco consumption was related to the risk of oral cancer, while patients suffering from oral cancer with at least one 5G allele of PAI-1 gene had a low risk for the development of clinical stage III or IV and lymph node metastasis compared with those with 4G/4G homozygotes in Taiwanese population	144
6	Tumor necrosis factor-α (TNF-α)	TNFA genetic variants (−308G>A and −238G>A) with the risk and prognosis of BQ-related	G allele and G/G genotype at TNFA −308 were associated with increased risk of cancer as compared to those with A allele or A/A+A/G genotypes. In addition, G allele and G/G genotype at TNF-α - 238 were associated with a borderline but statistically significant increased risk oral and pharyngeal squamous cell carcinoma (OPSCC) in Taiwanese population. Interactions between combined genotypes and smoking status were also found to contribute to risk of BQ-related OPSCC	145
7	Metallothionein 1 (MT-1)	rs8052394, rs11076161, rs8052334, rs964372, rs7191779 and rs708274 genotypes of MT-1	Individuals within Taiwanese population who inherited the MT-1 rs11076161 AA, rs964372 CC, and rs7191779 GC genotypes experienced significant protection against OSCC, whereas individuals carrying the MT-1 rs8052394 An allele seemed exposed to higher risk	146
8	N-acetyl transferase 2 (NAT-2)	Genetic polymorphism in NAT-2 resulting in slow NAT-2 acetylation haplotypes	The genotypic and allelic type of T341C and C481T in NAT-2 are associated with the risk of OPSCC in Taiwanese population	147
9	Glutathione-S-transferase (GST) genes	Polymorphism of GSTT1 gene	GSTT1 null genotype was found to be a significant risk factor for oral as well as gastric cancer in tobacco and BN associated cancer patients from Assam region of NE India	148
10	Microsomal epoxide hydrolase 1 (EPHX1)	139His/Arg genotype and 139Arg/Arg genotype	The 139His/Arg genotype was a significant risk factor for esophageal cancer in tobacco chewers and BQ chewers, while patients with the 139Arg/Arg genotype were at significantly higher risk for developing a well differentiated and moderately differentiated grade of tumor in India	149
11	hoGG1	Single nucleotide polymorphism (SNP) of hOGG1, codon 326	C allele of hOGG1 codon 326 may have a joint effect with BQ chewing on the development of oral cancer in Taiwanese population	150
12	Lysyl oxidase gene, LOX	G to A polymorphism at nucleotide 473 causing a non-conservative Arg158Gln change in the LOX amino acid sequence	The South Asian male patients of OSF older than 50 years had increased Arg158Gln in LOX	151
13	MDM2	Single nucleotide polymorphism in the MDM2 promoter (SNP 309)	The MDM2 SNP 309 GG genotype with mutated p53 contribute to early onset of both sporadic and hereditary malignancies in Taiwanese patients of BN associated OSCC	152
14	XRCC 4 intron 3	Ins/del variant	In smoker and BQ chewer groups, the XRCC4 intron 3 deletion variants exhibited 2.57- and 3.03-fold higher risks than the insertion genotype, respectively, in Taiwanese population	153
15	Cyclooxygenase (COX)	Polymorphisms of COX-2 −765G>C	COX-2 −765C allele vs. −765G/G genotype was a protective factor against OSCC development but was a risk factor for malignant potential of OSF in Taiwanese population	154
16	Matrix metallo-proteinase-9 (MMP-9) promoter	1562 C-to-T polymorphism	Enhanced OSCC risk in young Taiwanese male BN chewers	155
17	Matrix metallo-proteinase-3 (MMP-3) promoter	Insertion/deletion (−1171 5A–>6A) polymorphisms	5A genotype polymorphism - enhanced risk of OSF but not OSCC among male Asian BN chewers	156
18	NFκB1 promoter	Insertion/deletion polymorphism (−94 ins/del ATTG) in NFκB1 promoter	NFκB1 insertion and HO-1 L allelotypes – significantly enhanced risks for different subsets of OSCC in male Asian BN chewers	157
19	DNA repair genes *XRCC1* and *XPD*	Polymorphisms Arg194Trp, Arg280His, and Arg399Gln of the XRCC1 gene and Lys751Gln of the XPD gene	Variant allele of XRCC1 399 codon and XPD – enhanced risk of OC among South Indian BQ chewers and smokers	158
20	Heme oxygenase-1 (*HO-1*)	Polymorphisms in a (GT)n microsatellite repeat in HO-1 promoter in short (S), medium (M) and long (L) alleles	Longer (GT)n repeat allele L – higher risk of BN related OSCC; (GT)n repeat allele S - may be protective for OSCC in Asian population	159
21	Cytochrome gene *CYP2A6*	CYP2A6*4C mutation-gene deletion type of polymorphism	Deficient *CYP2A6* activity due to deletion – reduced risk of oral cancer risk in Sri Lankan BQ chewers	160
22	Cytochrome gene *CYP1A1*	*CYP1A1 A*/*G* genotype (Ile/Val) and *G*/*G* genotype (Val/Val) in exon 7	*CYP1A1* exon 7 containing *G* allele - enhanced risk for OSCC and oral precancerous lesion in Chinese BN chewer and smoker	161
23	Collagen related genes: Collagen 1A1 and 1A2 (COL1A1 and COL1A2), Collagenase-1 (COLase), transforming growth factor β1 (TGF-β1), Lysyl oxidase (LYOXase), and Cystatin C (CST3)	Polymorphisms of six collagen related genes, COL1A1, COL1A2, COLase, TGF-β1, LYOXase and CST3	Multigenic mechanisms involving the collagen related genes enhance susceptibility to OSF among Taiwanese BQ chewers	162
24	Tumor necrosis factor-α (TNF-α)	Bi-allelic promoter region (−308) polymorphism on the TNFα gene	The high production allele, TNF2 - significantly lower among individuals with OSF in Taiwanese population	163
25	Glutathione-S-transferase genes GSTM1 and GSTT1	GSTM1 and GSTT1 null genotypes (GSTM1*2 and GSTT1*2)	Null genotypes of either or both GSTM1 and GSTT1 - enhanced risk of development of leukoplakia following exposure to tobacco with or without BQ in South Indian population	164
26	Glutathione-S-transferase genes GSTM1 and GSTT1	Genetic polymorphism of GSTM1 and GSTT1	Homozygous deletion of GSTM1 gene – enhanced risk for oral cancer, which is further compounded by exposure to cigarette smoke, alcohol, and BQ in Thai population	165

### 10. BETEL NUT EXTRACT AND ROLE OF CYCLOOXYGENASE-2

Cyclooxygenase (COX), an inducible enzyme responsible for prostaglandin synthesis, plays an important role in certain inflammatory diseases and carcinogenesis. Tang *et al.* reported that COX-2 protein as well as mRNA expression was significantly enhanced in OSCC as compared to non-cancerous matched tissue (NCMT). Hydroxychavicol, a unique ingredient in BQ, also induced COX-2 overexpression in NHOK, indicating the early involvement of COX-2 in BQ associated OC [166]. Tsai *et al.* also reported that in human BMF, COX2 mRNA increased in a manner dependent upon increase in the dose of arecoline [167]. In addition, pretreatment with the GSH precursor, 2-oxothioazolidine-4-carboxylic acid, led to a decrease in the induction of COX2 mRNA by arecoline and the GSH synthesis inhibitor, buthioninesulfoximine, led to an increase, suggesting that regulation of COX2 expression induced by arecoline is critically dependent on cellular glutathione concentration [167]. Elevated COX2 protein levels have also been detected by immunohistochemistry in human tissues with moderate submucous fibrosis [9]. BNE was also found to induce COX2 mRNA and protein expression and PGE2 and 6-keto- PGF1α in primary HGK cells [168]. It was suggested that this stimulation of PGE2 production could partly result from the upregulation of COX2 mRNA expression. BN extract also slightly enhanced the activity of COX in the human oral carcinoma cell line, OEC-M1, but inhibited its activity in KB cells at concentrations greater than 50 µg/ml after 24 hours of exposure [169]. Upon treatment with BNE, head and neck carcinoma cells showed an increase of vimentin. The activation of extracellular signal-regulated kinase (ERK)/cyclooxygenase (COX)-2/prostaglandin (PGE)-2 cascade underlay the upregulation. These cells also exhibited the enhancement of migration and invasion. By knocking down COX-2 and vimentin expression, the increase of cell mobility was reversed. Tumors exhibiting extensive vimentin and/or COX-2 expression displayed a significantly worse disease-associated survival than contrast groups. The study thus revealed that BN-modulated vimentin expression enhanced the progression of head and neck carcinoma [170]. In another study, OECM-1 and Fadu cells developed a fibroblastoid morphology and there exhibited an increase in vimentin expression after BNE treatment. The treatment also induced the phosphorylation of AKT and glycogen synthase kinase 3β in OECM-1 cells. Blockage of phosphatidylinositol 3-kinase (PI3K)/AKT signaling attenuated vimentin expression when it was induced by BNE. However, it did not affect BNE-mediated extracellular signal-regulated kinase (ERK) activation or cyclooxygenase 2 (COX-2) upregulation. Oral carcinoma tissue samples were found to have significantly higher levels of vimentin and pAKT expression than their controls. Tumors exhibiting no vimentin expression and weak AKT phosphorylation were found to be associated with better survival than groups with higher levels of expression. These results imply that PI3K/AKT activation and vimentin expression are important pathogenic cascades in BN associated OC [171]. It thus appears that there is conclusive evidence to support a role of increased expression of vimentin in BN associated carcinogenesis [170], [171]. Involucrin is a key component of the cornified envelope and a differentiation marker of keratinocytes. Non-toxic BNE treatment of normal human oral keratinocyte (NHOK) was reported to induce a downregulation of involucrin and disruption of involucrin distribution and activation of AKT as well as upregulation of COX-2. The BNE associated downregulation of involucrin through AKT pathway could underlie the BN-associated epithelial pathogenesis [172].

### 11. BETEL NUT IN APOPTOSIS AND AUTOPHAGY

Autophagy is a regulated self cannibalism, classified as type II programmed cell death, and is preceded by the inhibition of the mammalian target of rapamycin (mTOR). The hallmarks of autophagy are the cleavage of the precursor form of microtubule associated protein 1 light chain 3 (LC3-I) (molecular weight = 18 kDa) to the active form LC3-II (molecular weight = 16 kDa) and the emergence of autophagic vacuoles (AV) and acidic vesicles [173]. Liu *et al.* reported that BNE induced (a) rounding cell morphology and nuclear shrinkage in different types of carcinoma cells, (b) the cleavage of LC3-I, and (c) the emergence of AV and acidic vesicles [173]. On the other hand, arecoline triggered (a) caspase-3 activation, (b) perinuclear chromatin condensation and (c) micronucleation, thus inducing atypical apoptosis. This difference is thought to be due to the ability of BNE, but not arecoline, to inhibit the phosphorylation of the mTOR-Ser2448. Lu *et al.* reported that BNE treatment induced autophagy among oral cancer cells characterized by LC3-II accumulation, genesis of autophagosomes and the appearance of EGFP-LC3 puncta [173]. Significantly, the blockage of BNE induced autophagy increased the proportion of oral cancer cells undergoing apoptotic death indicating that the eventual induction of autophagy was beneficial to cell survival from BNE induced apoptosis [174]. Transmission electron microscopy (TEM) of liver precancerous nodules induced in Swiss Albino mice upon transgenerational exposure to AEBN revealed enhanced cristolysis of mitochondria and formation of AV [132]. Cristolysis would induce deficiency of oxidative ATP production and inhibition of apoptosis. Moreover, the cells would have to meet their nutritional requirements through autophagy, thus, surviving and proliferating in the face of metabolic stress.

### 12. BETEL NUT AND MALIGNANT LESIONS

Studies and follow-up programmes conducted over the past three decades indicate that the rate of malignant transformation of pre-cancer lesions shows a population dependent variability. A study conducted in Karachi, Pakistan (1996–98) on 79 cases of OSCC and 149 hospital controls showed that the risk for developing OC was 19 times higher (95% CI, 4.2–87.7) among cases of OSF than among subjects with no precancerous condition [175]. In a study by Shiu *et al.* conducted in Taiwan, 60 cases of oral and pharyngeal cancers, including lip, tongue, gum, mouth floor, buccal palate, oropharynx and hypopharynx cancers were evaluated by linking a retrospective leukoplakia cohort consisting of 435 patients recruited from hospitals between 1988 and 1998 to a population cancer registry [176]. The risk for malignant transformation increased with time, particularly for BN chewers. Using a Weibull survival model, the adjusted hazard ratio for chewing BN without tobacco was 4.6 (95% CI, 1.3–16.9) after matching for age and sex.

Thus, the presence of pre-cancer lesions appears to correlate well with malignant transformation and eventual development of OC (Figure 5C). Besides OC, individuals who chewed BN or BQ without or with tobacco also reported other aerodigestive cancers. From 1997 to 1998, a hospital based case–control study on oesophageal cancer in Assam, India, included 502 cases (358 men, 144 women) and 994 controls (706 men, 288 women) who were attendants to cancer patients. The risk for esophageal cancer increased with increasing frequency of chewing BQ without or with tobacco and increased substantially when the chewing habit had lasted 20 years or more. A dose–response relationship was also observed for age at starting the habit, with a higher risk for starting at a younger age [177].

### 13. EFFORTS TO CONTROL USE OF BETEL NUT, BETEL QUID AND ADDITIVES, INCLUDING *PAAN MASAL* AND *GUTKHA*

It is a scientifically established fact that BN or BQ without or with various additives, including tobacco products, are clearly associated with increased risk of HNC, especially OC. In spite of this fact, it has remained a challenge for governments and policy makers to contain its usage and reduce the deleterious effects of BN or BQ on population due to its socio-cultural heritage and addictive nature. Global data on efforts to control usage of BN or BQ and its additives strongly suggest that no single strategy is likely to succeed in achieving the desired level of control on BN or BQ consumption. A concerted effort involving radical measures is required in this direction. It may involve health care professionals, media, policy makers, law enforcing agencies and the community at large. Even though no unified guideline exists due to variation in the socio-cultural groups, it appears practical to make sustained behavioral interventions, especially adolescents and young adults, for reducing the use of BN or BQ without or with additives. Lack of scientific information on its deleterious effects on health is conspicuous by its absence. In contrast, various advertisements try to glorify the opposite in order to allure most vulnerable section of the society, the adolescents and young adults. Once addicted, they become a long term consumers of BN or BQ. Media may play a very important role in this effort due to its widespread reach in all strata of the society, particularly the lower strata, which appears to be deeply affected by this practice. Governmental and non-governmental health care professionals may routinely assess and record the usage pattern in the society and rigorously inform the individuals, groups of people or patients about its potential hazard. Chang *et al.* reported that an oral screening program conducted in a tertiary medical centre for detection of oral lesions and oral cancer was effective. The group suggests that individuals aged ≥40 years or who are habitual cigarette smokers, alcohol consumers, and BQ chewers should receive oral screening regularly so that potential oral cancer can be detected as early as possible [178].

Unnecessary glorification of the BN or BQ products in media must be stopped by enacting appropriate laws. Visual representation of the diseases, particularly OC, caused by the use of these products and appropriate warning signs may be effective in this campaign. The industrial policies, including taxation, need revisit in order to limit such industrial activities to discourage marketing of such products. There is need to have strong political commitment towards this aim. Many countries have made some headway in this direction. A brief summary of such efforts is listed below:

#### (a) India

On 1 August 2002, the Commissioner for Food and Drug Administration and Food (Health) Authority, Maharashtra State, issued a gazette notification banning the manufacture, sale and storage of *gutkha* and *paan masala* or any similar product containing or not containing tobacco. In India, a warning label is now mandatory on packets of commercial BN and tobacco products, but there are no regulations about the size of the letters. Several states are at various stages of passing laws to ban *gutkha* or are in court after being challenged by the industry. A recommendation that *gutkha* should be banned nationwide has been made to the Government of India by the Central Committee on Food Safety [9]. Recently, a ban on using plastic packing was passed by the Supreme Court of India with effect from March 2011 and is being implemented at the time of writing this review (http://www.tobaccojournal.com/Chewing_tobacco_plastic_pouches_banned.50432.0.html). On the occasion of World No-Tobacco day on the 31^st^ May 2012, the state of Bihar joined two other states (Kerala and Madhya Pradesh) in banning manufacture, storage, distribution and sale of any form of tobacco and nicotine containing *paan masala* and *gutkha* for a period of 1 year to begin with (http://indiatoday.intoday.in/story/bihar-bans-sale-of-gutkha-for-a-year/1/198280.html).

#### (b) North America

BN figures on the list of herbs that are unacceptable as a non-medicinal ingredient in oral use products. The sale of BN products has been banned in Canada as a result of the link between arecoline and mutagenic effects. The US FDA maintains an import alert within the USA, the main concerns being adulteration and addition of unsafe food additives. In 1976, the US Government announced a ban on interstate traffic of BN [9].

#### (c) European Union

Within the European Union, excluding Sweden, there is legislation banning the sale of tobacco products for oral use. However, there are no specific laws regulating or banning the sale of BN products, even when mixed with smokeless tobacco, as chewing tobacco is excluded from the directive [9].

#### (d) United Kingdom

In the United Kingdom, there is no law to regulate the import or sale of products containing BN. Presently numerous BN preparations, with or without tobacco, are commercially available in shops. The Department of Trade and Industry classifies these products as sweets. Labelling and a list of ingredients on the packaging are sometimes non-existent. Several studies have shown that in most outlets the sales are unrestricted to minors and children under the age of 16 were able to purchase *gutkha* easily. Only a few BN products give specific health warnings on the dangers of chewing BN, although most carry the statutory health warning regarding added tobacco. Among 20 commercially processed and packaged BN products on sale in the United Kingdom, only three carried a health warning related to OC; none warned about OSF or potential addiction [9].

#### (e) Other Countries

In the late 1970s, the Public Services of Papua New Guinea issued a ban on BQ chewing in government offices. Possession of BN in the California public school system is grounds for suspension. In Singapore, spitting in public places can lead to a fine, indirectly discouraging the practice of BQ and BN chewing [9].

## Conclusions

BN and products derived from it are widely used as a masticatory among various communities, and in several countries across the world, as a socially endorsed habit [1]–[11]. Over a long period, several additives got added to a simple BN preparation, thus, creating the BQ and encompassing chewing tobacco in the preparation. The addictive nature of BN and/or the additives that make it BQ, are essentially responsible for its rampant usage among individuals [19]–[22]. The popularity has lead to industrial preparation of convenient substitutes of the BN/BQ in the form of *paan masala*, *gutkha* and the likes in several countries. This review reveals that BN has far reaching consequences on the general health of the masticator, especially in context with the oral health of the users [23]–[56]. Extensive studies by several workers over the years conclusively prove the role of BN, and its components, primarily the alkaloid arecoline, as a carcinogen [55]–[61]. These substances not only have general mutagenic, cytotoxic and genotoxic properties, but are also intricately involved in enzymatic, molecular and genetic mechanisms that result in the development of carcinogenesis at various sites, specifically in the oral cavity [58]–[150]. More research in clearly required to fill many existing gaps in the understanding of the seemingly highly complex interactions of BN with the life process and its manifestation in HNC, particularly OC. Control over human consumption of BN and BQ without or with additives, including tobacco, or its convenient commercial substitutes, such as *gutkha* and *paan masala*, is proving to be difficult because the habit is not associated with any social stigma and taboo. In fact, it is other way round in which this practice has largely been given social, religious or other sanctions in different regions of the world. Hence, strong multifaceted intervention is required to discourage or control the habit of BN/BQ mastication. Firstly, legislation against open sale and use of such products should be stricter and more states and countries should bring out such legislations sooner than later. Secondly, public awareness should be created regarding the harmful effects of these products among all sections of society, particularly among children, since the habit starts early in the majority of cases. Lastly, attempts need to be made for harm-reduction of BN/BQ/commercial substitutes. Multi-institutional transnational case controlled studies are also required in order to establish the exact etiopathogenesis and molecular changes of diseases caused by BN/BQ and/or its constituents.
